# G-computation and machine learning for estimating the causal effects of binary exposure statuses on binary outcomes

**DOI:** 10.1038/s41598-021-81110-0

**Published:** 2021-01-14

**Authors:** Florent Le Borgne, Arthur Chatton, Maxime Léger, Rémi Lenain, Yohann Foucher

**Affiliations:** 1 INSERM UMR 1246 - SPHERE, Nantes University, Tours University, 22 Boulevard Bénoni Goullin, 44200 Nantes, France; 2 IDBC-A2COM, Pacé, France; 3 Département D’Anesthésie Réanimation, Centre Hospitalier Universitaire D’Angers, Angers, France; 4 Lille University Hospital, Lille, France; 5 Nantes University Hospital, Nantes, France

**Keywords:** Medical research, Epidemiology

## Abstract

In clinical research, there is a growing interest in the use of propensity score-based methods to estimate causal effects. G-computation is an alternative because of its high statistical power. Machine learning is also increasingly used because of its possible robustness to model misspecification. In this paper, we aimed to propose an approach that combines machine learning and G-computation when both the outcome and the exposure status are binary and is able to deal with small samples. We evaluated the performances of several methods, including penalized logistic regressions, a neural network, a support vector machine, boosted classification and regression trees, and a super learner through simulations. We proposed six different scenarios characterised by various sample sizes, numbers of covariates and relationships between covariates, exposure statuses, and outcomes. We have also illustrated the application of these methods, in which they were used to estimate the efficacy of barbiturates prescribed during the first 24 h of an episode of intracranial hypertension. In the context of GC, for estimating the individual outcome probabilities in two counterfactual worlds, we reported that the super learner tended to outperform the other approaches in terms of both bias and variance, especially for small sample sizes. The support vector machine performed well, but its mean bias was slightly higher than that of the super learner. In the investigated scenarios, G-computation associated with the super learner was a performant method for drawing causal inferences, even from small sample sizes.

## Introduction

Machine learning (ML) is a set of mathematical and statistical methods that computer systems use to perform tasks without specific instructions. In medical research, there is an increasing interest in these methods for prediction and, more recently, for causality^1^ There is a large intersection between these fields since the first step of causal modelling consists of predicting the exposure for propensity score (PS)-based methods^2,3^ or the outcome for G-computation (GC)^4,5^.

Several recent methodological studies have therefore studied the potential applicability of ML for causal inference. A large number simulation-based studies have compared several ML methods to obtain PSs^1,6–10^. While the corresponding PS-based results were very encouraging, GC was compared to PS-based methods in the context of classical regression models and showed several advantages in terms of statistical power^11–14^ and robustness of the estimates regardless of the set of included covariates^11^. However, simulation-based studies related to the use of ML for predicting outcomes in GC are infrequent. Austin examined the use of ensemble-based methods (bagged classification and regression trees (CART), random forests, and boosted CART (BCART)) and concluded that BCART was the highest performing algorithm^15^. He also concluded that BCART had a lower bias when it was used to impute potential outcomes than when it was used to estimate the PS for inverse probability treatment weighting.

In this paper, we studied the performances of GC in combination with different ML algorithms, including a super learner (SL), through simulations to estimate causal effects. Many of the previous studies were based on large samples. Therefore, we made sure to include scenarios with small sample sizes. We limited our study to case where both the exposure and outcome were binary and to small-medium sample sizes. We also focused on ML techniques that are applicable in daily practice, i.e., with reasonable computation times on modern laptops or workstations.

## Methods

### G-computation

Let $Y\left(1\right)$ and $Y\left(0\right)$ be the two potential outcomes under the exposure and the non-exposure, respectively^16^. Let $\left(Z,X\right)$ denote the random variables related to the exposure statuses of individuals ($Z=1$ for exposed individuals and $0$ otherwise) and the $k$ covariates ($X={X}_{1}, \dots , {X}_{k}$) measured before exposure, respectively. The average causal effect is $ACE = E\left[Y\left(1\right)-Y\left(0\right)\right]$. It represents the mean difference between the outcomes of individuals if they had been exposed or unexposed^17^.

Suppose $\left({Y}_{i}, {Z}_{i}, {X}_{i}\right)$ a dataset for analysis consists of $n$ independent realisations of $\left(Y, Z, X\right)$. The first step of GC is to fit $f\left(Y|Z,X\right)$, and this outcome model is frequently referred to as the Q-model^5^. Once estimated, the Q-model aims to predict, for each individual $i$ ($i=1,\dots ,n$), the two potential outcomes under each exposure status by maintaining her/his covariates ${X}_{i}$ at the observed values and setting ${Z}_{i}$ to 1 and 0: ${\widehat{Y}}_{i}\left(1\right)= \widehat{f} \left(Y|1,{X}_{i}\right)$ and ${\widehat{Y}}_{i}\left(0\right)= \widehat{f} \left(Y|0,{X}_{i}\right)$. The average causal effect is then estimated by $\widehat{ACE} ={n}^{-1} {\sum }_{i=1}^{n}\left[{\widehat{Y}}_{i}\left(1\right)-{\widehat{Y}}_{i}\left(0\right)\right]$.

### Covariates selection

One of the main differences between prediction and causality is the selection of covariates. Knowledge of the causal relationship structure is essential for conducting causal inference^18^. This knowledge consists of excluding the mediators, colliders^19^, and instrumental variables^20,21^. Note that a benefit of GC over PS-based methods is that it more effectively prevents instrumental variables, which are often included in the PS. In this context, the advantages and limits of ML algorithms have been well described^22,23^. As noted by VanderWeele and Shpitser^24^, investigators can identify the causes of exposure statuses or outcomes as potential covariates.

Unfortunately, full knowledge of causal relationships is often unavailable. There is a growing literature about the best set of covariates to consider, and it recommends including all the covariates that cause the outcome^11,21,25^. The corresponding data-driven selection procedure for GC is straightforward since it corresponds to the predictors of the Q-model.

### ML techniques

In contrast with PS-based methods, which consist of predicting exposure statuses, the Q-model must keep the exposure status as one of the predictors. This is not possible for several ML techniques, such as random forests, except by estimating $f(.)$ separately for the exposed and unexposed individuals. Nevertheless, this solution is not reasonable for small sample sizes (we have tested it, and the results confirm its deficient performances for $n < 1000$; data not shown). Below, we briefly describe the ML methods that we included in our simulations. For more details on these ML techniques, see McNeish for the penalized methods^26^, and Bi et al. for the other methods^27^. We performed all the analyses using R version 3.6.1.

#### Lasso logistic regression (LLR)

L1 regularisation allows for the selection of the predictors. To obtain a flexible model, we considered all the possible interactions between the exposure status $Z$ and covariates $X$. Moreover, we used b-splines for the quantitative variables of the vector $X$. We used the *glmnet* function included in the *glmnet* package.

#### Elasticnet logistic regression (ELR)

We used the same flexible logistic regression as previously defined, but with both the L1 and L2 regularisations (two tuning parameters).

#### Neural network (NN)

We chose a neural network with one hidden layer, as this is probably the most common network architecture^3^. Its size constitutes the single tuning parameter. We used the *nnet* function of the *nnet* package.

#### Support vector machine (SVM)

We chose the radial basis function kernel to flex the linear assumption. We used the *svmRadial* function of the *kernlab* package with two tuning parameters: the cost penalty of misclassification and the flexibility of the classification.

#### Boosted CART (BCART)

This ML technique is an ensemble method, that is, a method that averages the percentages of events in the terminal nodes of several tree partitions. Four tuning parameters must be chosen: the number of trees, the highest level of covariate interactions, the learning rate, and the minimum number of observations in the terminal nodes. We used the *gbm* function included in the *gbm* package.

For the five methods listed above (LLR, ELR, NN, SVM, and BCART), we chose their respective tuning parameters by maximising the average area under the receiver operating characteristic curve (AUC) of tenfold cross-validation. We used the *caret* package with a tuning grid of length equals 20.

#### Super learner (SL)

We included the previous ML techniques in the SL, with the exception of BCART due to the resulting computational burden. The SL consists of averaging the predictions obtained from the four approaches by using a weighted linear predictor^28^. In agreement with our previous choice, we estimated the weights by maximising the average AUC of tenfold cross-validation. We used the *SuperLearner* package.

### Variance estimation

By bootstrapping the entire procedure^29^, one can obtain the standard error and the confidence interval of the $ACE$. Regarding the corresponding computational burden, a compromise consists of choosing the tuning parameters based on the entire sample and then using these values in the subsequent bootstrap samples^30,31^. Moreover, to consider the possible overfitting associated with such ML techniques, we performed a bootstrap cross-validation procedure. We trained the ML algorithms from the bootstrap sample, while we estimated the $ACE$ from the individuals not included in the bootstrap sample. In this paper, we performed 500 iterations.

## Simulation-based study

### Data generation

**Figure 1:**
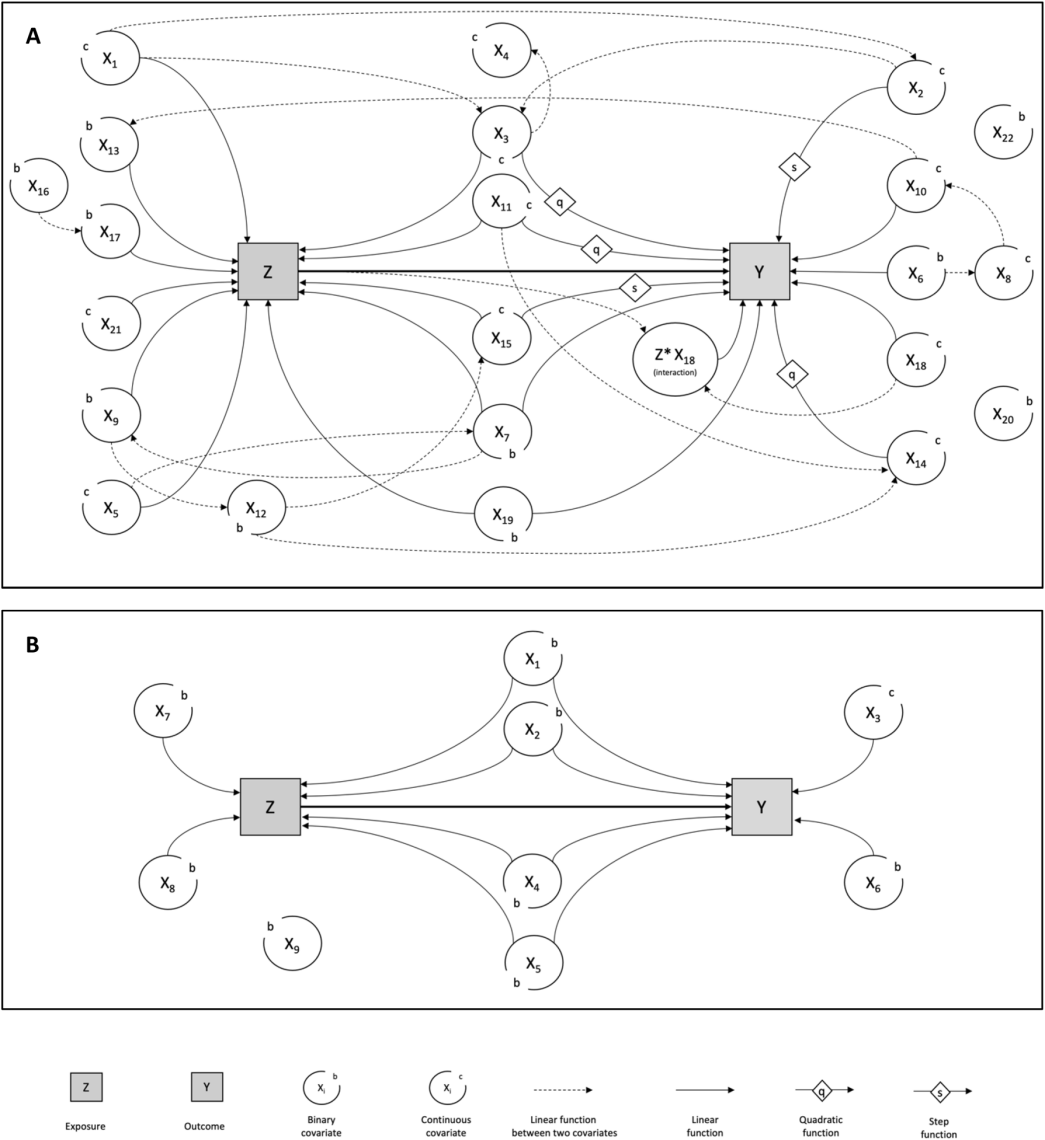
Directed acyclic graphs associated with the two simulated scenarios. (**A**) The realistic scenario with 22 covariates, linear and nonlinear relationships, and one interaction. (**B**) The simplistic scenario with nine covariates, linear relationships, and no interaction.

We considered two main scenarios, as illustrated in Fig. 1 (the related models are in Supplementary Tables S1 and S2). First, we simulated the continuous and binary covariates from ${X}_{1}$ to ${X}_{k}$, allowing for dependences between the simulated covariate and those already generated. Second, we obtained $Z$ and $Y$ with Bernouilli distributions. The logit of the corresponding probabilities equaled the linear functions of $X$ and ($X,Z$).

We choose two contrasting scenarios. We defined a realistic situation (Fig. 1A, Supplementary Table S1) with 22 correlated covariates at baseline. Nine covariates were included in the outcome model, among which one covariate interacted with the exposure effect, two effects were step functions, three were quadratic functions, and four were linear. In contrast, we defined a simplistic situation (Fig. 1B, Supplementary Table S2) with nine independent covariates. Six covariates were included in the outcome model with linear effects and no interaction.

We simulated all the covariates $X$ as variables measured before exposure. We did not consider mediators and colliders. As previously stated, the investigator must exclude these variables from the set of covariates. We studied different sample sizes: *n* = 100, 500, and 1000. For each scenario, we randomly generated 10,000 datasets.

### Performance criteria

We computed the theoretical $ACE$ by averaging the $ACE$ estimations obtained from the univariate logistic models (with $Z$ as the only explanatory variable) fitted based on datasets that were simulated as above, except that $Z$ was generated independently of $X$^11,32^. We reported the following criteria (the formulae can be found in the Supplementary Materials): the mean bias (MB), the root mean square error (RMSE), the empirical standard deviation (ESD), the asymptotic standard deviation (ASD), the variance estimation bias (VEB), the empirical coverage rate of the nominal 95% confidence interval (95% CI), and the statistical power. We compared the performances of the previous ML techniques. In addition, we examined the results and compared them with those obtained by a perfectly specified LR, i.e., a LR with the same linear predictor as the one defined in the last lines of Supplementary Tables S1 and S2, in which we only estimated the corresponding regression coefficients.

### Comparison of the ML techniques in terms of bias

#### Overall results

To evaluate the calibration of the ML methods for the simulated data, we added calibration plots of 10 simulated datasets for each combination of methods (LLR, ELR, NN, SVM, SL), complexity (simplistic, realistic), and sample size ($n=100, 500, 1000$) to the Supplementary Materials (Figures S1-10). One can observe an overfitting of the ELR, SVM, and SL when $n=100$, and this can be explained by the fact that the number of parameters was too large compared to the sample size.

**Figure 2:**
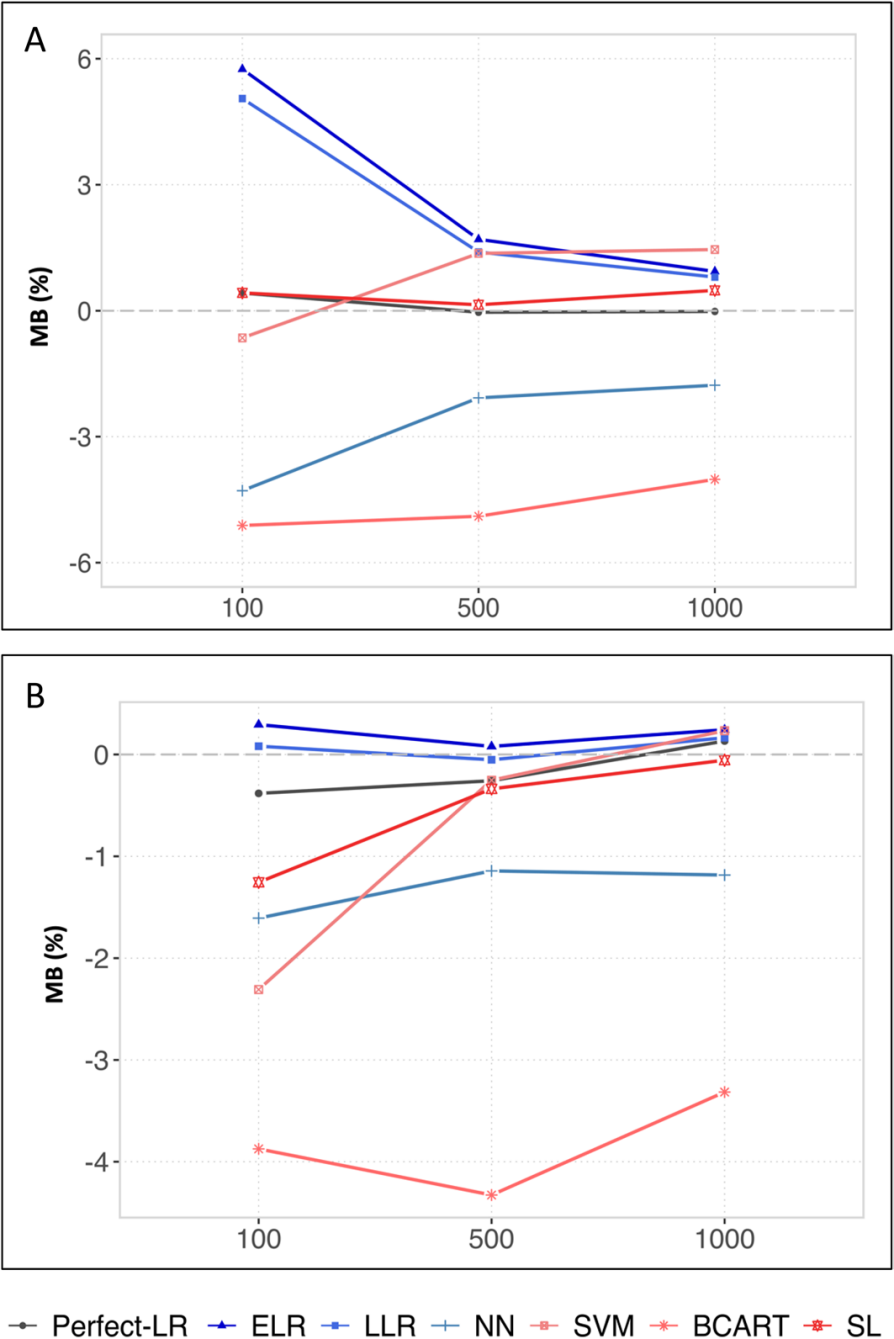
Mean biases (MBs) of G-computation in realistic (**A**) and simplistic (**B**) situations with the following Q-models: the theoretical logistic regression, elasticnet logistic regression, lasso logistic regression, neural network, support vector machine, boosted CART and super learner.

**Figure 3:**
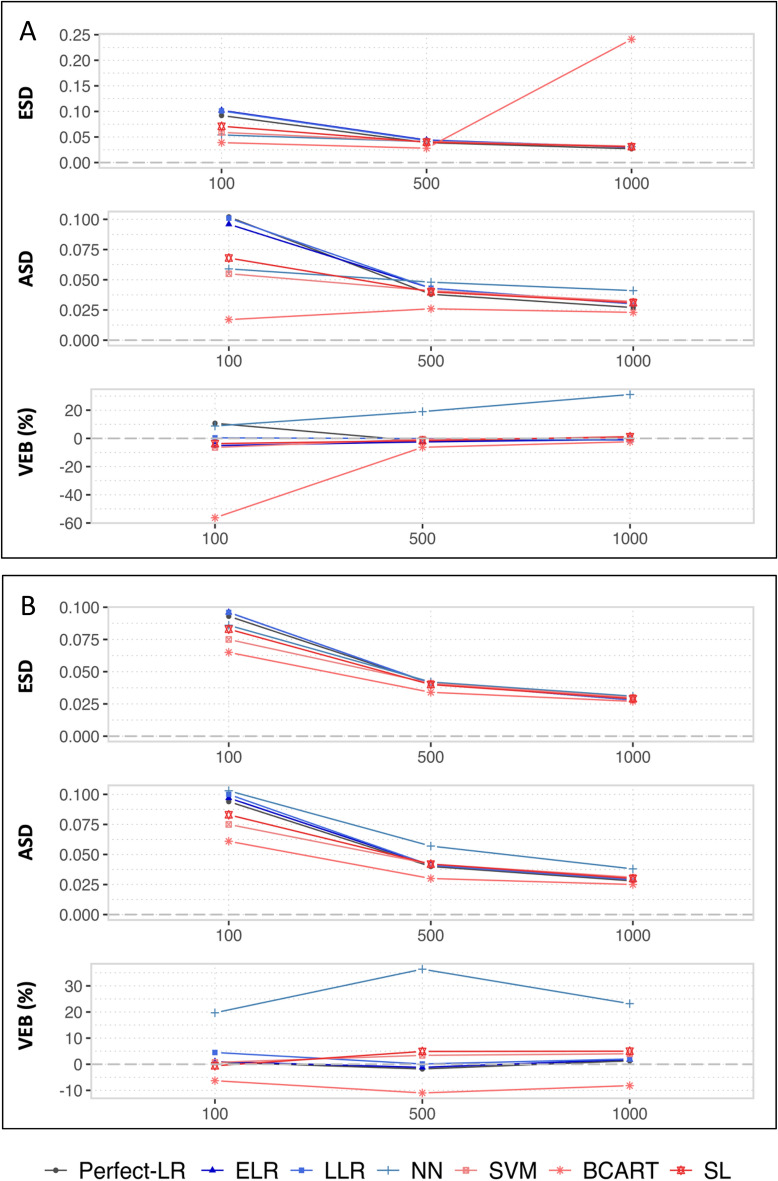
Empirical and asymptotic standard deviations **(**ESDs and ASDs, respectively) and variance estimation biases **(**VEBs) of G-computation in realistic (**A**) and simplistic (**B**) situations with the following Q-models: the theoretical logistic regression, elasticnet logistic regression, lasso logistic regression, neural network, support vector machine, boosted CART and super learner.

**Figure 4:**
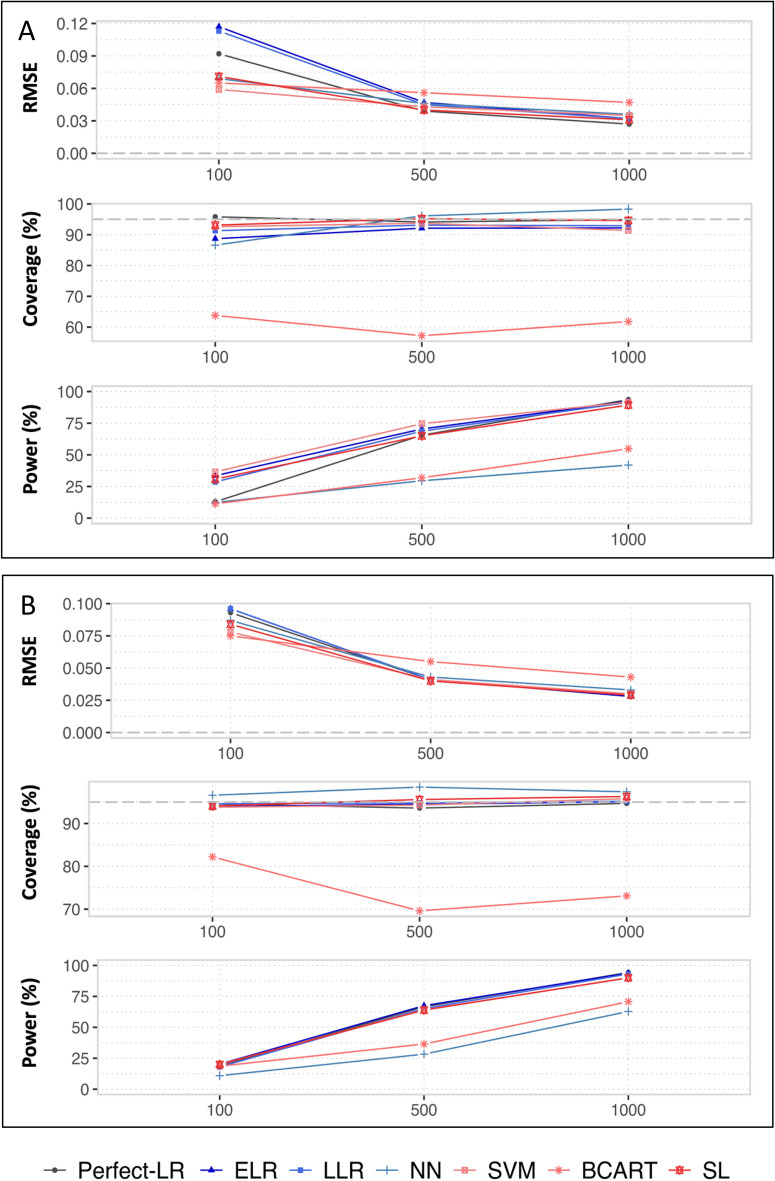
Root mean square errors (RMSEs), coverages and powers of G-computation in realistic (**A**) and simplistic (**B**) situations with the following Q-models: the theoretical logistic regression, elasticnet logistic regression, lasso logistic regression, neural network, support vector machine, boosted CART and super learner.

We report the simulation results in Figs. 2, 3 and 4 for the realistic and simplistic scenarios (the numerical details can be found in Supplementary Tables S3 and S4). Independent of the sample size and the complexity of the relationships between the covariates and the outcome, BCART was associated with a significant level of bias, with the MB being higher than 3%.

#### The impact of the sample size in the realistic situation

To differentiate between the other methods, one can compare the MBs obtained when the relationships between the covariates and outcome are difficult for the analyst to manage, i.e., a realistic situation. When the learning support is small ($n=$ 100), the penalized methods (ELR and LLR) and the NN resulted in unacceptable MBs higher than 4%. In contrast, the two remaining methods (SVM and SL) were associated with values lower than 1%. With large sample sizes ($n\ge$ 500), the four methods performed correctly with MBs less than 3%, and the lowest MB was obtained with the SL (MB < 1% for all sample sizes). To further discriminate between the SVM and SL in this realistic situation, one can notice that the MB remained negligible for the SL regardless of the sample size, while for the SVM, the MB increased with the sample size (values between 1 and 2% when $n\ge 500$).

#### The impact of the sample size in the simplistic situation

Except when $n=1000$, for which they were outperformed by the SL (MB < 1%), the penalized methods were associated with the smallest biases in the simplistic situation, with MBs less than 1% regardless of the sample size. The penalized methods were even the only methods such low values when $n=100$. The NN was the only method with no significant variations according to the sample size (i.e., MBs between 1 and 2% for all three sample sizes).

### Comparison of the ML techniques in terms of variance

#### Overall results

Regardless of the scenarios and the sample sizes used, one can observe an underestimation of the variance using BCART. Its VEB ranged from − 2 to − 56%.

#### The impact of the sample size in the realistic situation

To differentiate between the other methods, one can first consider the smallest sample size ($n =$100). The penalized approaches (LLR and ELR) resulted in the highest estimations of the variance, with ASDs close to 0.10. The SVM and NN were associated with the smallest variances, with ASDs close to 0.6 (the VEBs were − 6.4% and 8.8%, respectively). Compared with the two previous ML techniques, the SL resulted in a slightly higher ASD at 0.7, but a lower VEB at − 3.7%. For larger sample sizes (*n* ≥ $500$), the results in terms of variance were close for the four following approaches: LLR, ELR, SVM and SL. The NN was associated with an unacceptable overestimation of the variance (VEB = 19.0% and 31.1% for *n* = 500 and 1000, respectively).

#### The differences between the realistic and simplistic situations

The results were similar when the relationships between the covariates and the outcome were easier for the analyst to model (i.e., the simplistic situation). However, one can underline an exception: when *n* = 100, the NN resulted in an ASD close to those of the penalized approaches.

### Synthesis of bias and variance in terms of the root mean square error and coverage

Even if BCART resulted in a critical level of bias, its RMSEs were reasonable, and this is mainly because of the previously reported underestimation of the variance. This bias associated with an underestimated variance resulted in coverage ranging from 57.2 to 82.2%, and the upper bound of this range is considerably lower than the nominal value of 95%. For the smallest sample size, in both the realistic and simplistic situations, the RMSEs of the penalized methods were among the highest because of their high-level of variance (simplistic situation) or high levels of bias (realistic situation).

When *n* ≥ $500$, the RMSEs of the penalized methods were close to those observed for the ML-based methods (NN, SVM and SL). However, for these two approaches, one can observe slightly anti-conservative 95% CIs in the realistic situation, because of their slight biases. For the remaining ML-based methods, the RMSEs were comparable for the three sample sizes and in the two situations, but the results of the NN should be interpreted with caution. Indeed, for *n* = 100, the NN was associated with a significant bias, but a low variance estimation, resulting in a CI of 86.6%, lower than the nominal value of 95%.

As previously reported, the two remaining methods (SL and SVM) were the two ML techniques associated with the smallest MBs. For each scenario, the MB of the SL was even lower than the value of SVM. This explains why the nominal coverage was slightly higher when using the SL. For instance in the realistic scenario, the coverage values associated with the SVM were 92.6%, 93.7% and 91.4% for $n\hspace{0.17em}=\hspace{0.17em}$100, 500 and 1000, respectively, while they were 93.1%, 95.2% and 94.6% for the SL.

### Power of the unbiased methods

We only consider the methods and the scenarios in which the MB were lower than 1% due to the problems encountered when interpreting the power in the presence of bias.

#### The realistic situation

When $n\hspace{0.17em}=\hspace{0.17em}100$, the SVM and SL had MBs lower than 1%. Of the two methods, the best power was achieved by the SVM (36.5% vs 30.8% for the SL). When $n\hspace{0.17em}=\hspace{0.17em}1000$, the ELR, LLR and SL had MBs lower than 1%, and the best power values were achieved by the penalized methods (92.4% for the ELR, 91.5% for the LLR and 89.3% for the SL).

#### The simplistic situation

When $n\hspace{0.17em}=\hspace{0.17em}$100, only the penalized methods had MBs lower than 1%. The best power was obtained by the ELR (20.2% versus 18.0% for the LLR). When $n\hspace{0.17em}\ge \hspace{0.17em}500$, we additionally observed MBs lower than 1% for the SVM and SL. The penalized methods were always associated with the best powers when compared with those of the two ML techniques with a gain between 1 and 4% depending on the scenarios.

### ML techniques versus the perfectly specified LR

The performances of the perfectly specified LR were better than those of the ML techniques for large sample sizes ($n=\hspace{0.17em}$1000). One can observe mean bias values close to 0%, and variance bias values close to 1%. Nevertheless, when the sample size decreased in the realistic situation, the performances of the perfectly specified LR decreased more than those of several ML techniques. When $n=\hspace{0.17em}$500, the variance bias associated with the perfectly specified LR was − 2.1% versus − 0.1% for the LLR, − 0.5% for SVM and − 1.6% for the SL. When $n=\hspace{0.17em}$100, the variance bias associated with the perfectly specified LR was 10.7% versus 0.4% for the LLR, − 3.7% for the SL, and − 6.4% for the SVM. In this latter scenario, these three ML techniques resulted in higher statistical powers than the one obtained with the perfectly specified LR.

## Application

### Context

We applied the methods to evaluate the efficacy of barbiturates prescribed during the first 24 h of an episode of intracranial hypertension. The control group included patients without barbiturates at 24 h. One can use this treatment to decrease refractory intracranial pressure, but its effectiveness remains debated due to the associated adverse events (*e.g.*, haemodynamic impacts or infectious complications).

We used data from the French prospective cohort AtlanREA. We considered patients with intracranial pressures higher than 20 mmHg. We conducted this study following French law relative to non-interventional clinical research. Written informed consent was collected. Moreover, the French commission for data protection approved the collection (CNIL DR-2013-047). The study was approved by the AtlanREA scientific council (www.atlanrea.org) and the ethics committee of the French Society of Anesthesia and Intensive Care (SFAR, https://sfar.org/).

### Implementation of the methods

We reduced the set of covariates to the possible causes of the outcome without considering the consequences of barbiturate use. We described this selection in detail in Supplementary Table S5. For the ML-based methods, we considered all the covariates before exposure and the corresponding interactions with the exposure status. As in the previous simulations, we used b-splines for the continuous covariates in the penalized methods. For the investigator-based method, all the outcome causes previously listed were included (Supplementary Table S5). The log-linearity assumption for continuous covariates seemed to be satisfied. We assumed that there was no interaction because of the absence of clinical relevance.

## Results

**Figure 5:**
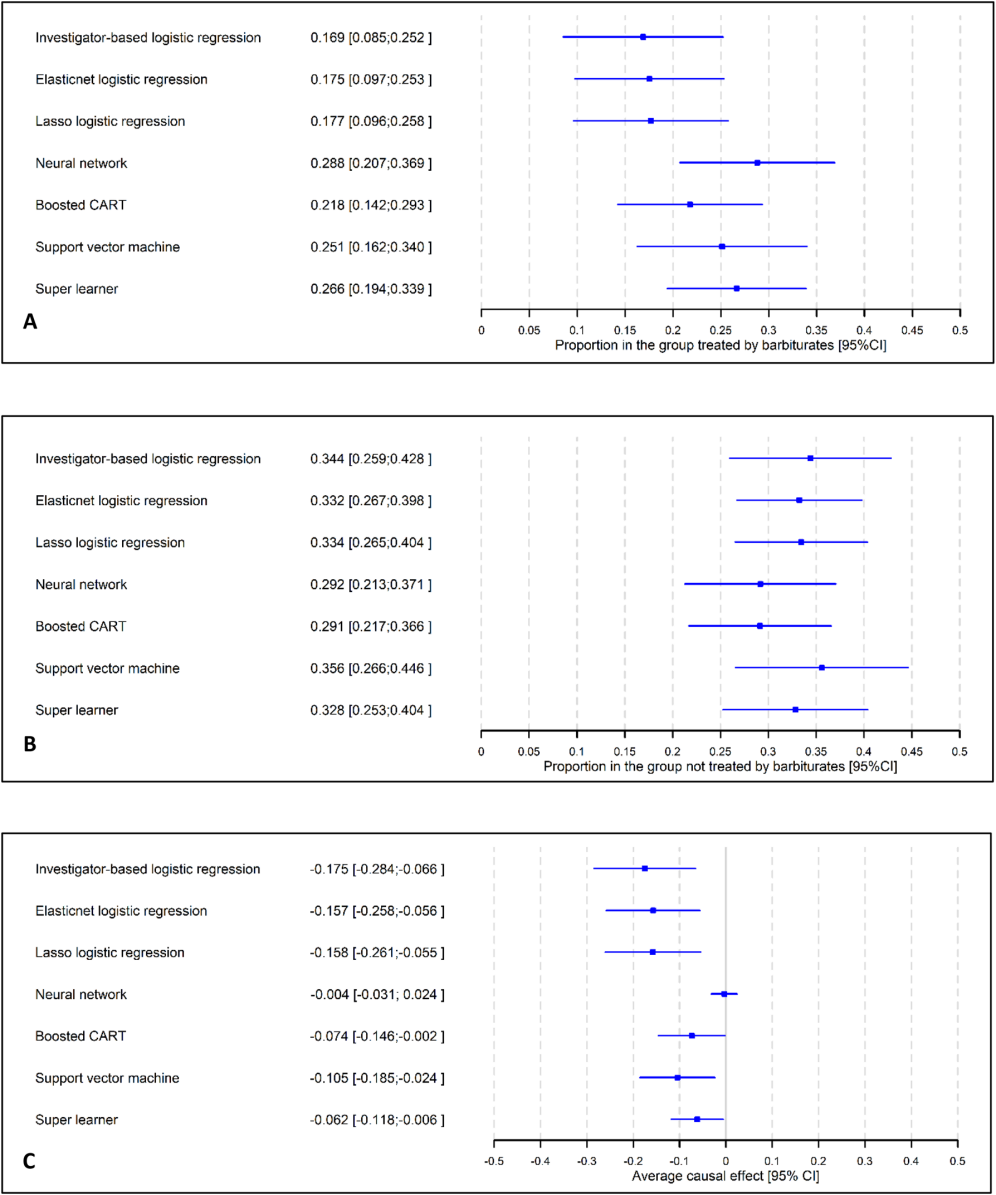
Estimations of the confounder-adjusted proportions of patients with favourable GOS among the patients treated with barbiturates (**A**), patients not treated with barbiturates during the first 24 h postadmission (**B**), and the corresponding average causal effects (**C**).

Table 1 describes the 252 patients. Seventy-four patients were in the treatment group. The outcome was the proportion of patients with a favourable Glasgow Outcome Scale (GOS ≤ 3) at three months after admission to the intensive care unit. Figure 5 presents the confounder-adjusted estimates. The investigator-based approach resulted in a 17.5% decrease in the percentage of patients with favourable 3-month GOS due to barbiturates (95% CI from 6.6 to 28.4%). We observed similar results for the ELR and LLR, in terms of both the estimates and the 95% CIs. The other ML techniques resulted in lower associations, and the one fpr the NN was even nonsignificant ($ACE=\hspace{0.17em}$0.4%, 95% CI from − 3.1 to 2.4%). The SL resulted in a small but significant association ($ACE=\hspace{0.17em}$6.2%, 95% CI from 0.6% to 11.8%).

**Table 1:** Baseline characteristics of patients according to the treatment group (*n* = 252) and the GOS at three months after the treatment initiation.

	Overall ($n$ = 252)		Barbiturates treatment					Favourable GOS at three months				
			No (*n* = 178)		Yes (*n* = 74)		*p*	No (*n* = 180)		Yes (*n* = 72)		*p*
Female patient (n, %)	89	35.3	58	32.6	31	41.9	0.1592	68	37.8	21	29.2	0.1963
Diabetes (n, %)	17	6.7	15	8.4	2	2.7	0.0989	15	8.3	2	2.8	0.1122
No sological entity: severe trauma (n, %)	124	49.2	95	53.4	29	39.2	0.0403	77	42.8	47	65.3	0.0012
SAP ≤ 90 mmHg before admission (n, %)	56	22.2	36	20.2	20	27.0	0.2368	46	25.6	10	13.9	0.0442
Evacuation of subdural or extradural hematoma (n, %) (*)	41	16.3	33	18.5	8	10.8	0.1301	27	15.0	14	19.4	0.3878
External ventricular drain (n, %)	64	25.4	39	21.9	25	33.8	0.0486	48	26.7	16	22.2	0.4640
Evacuation of cerebral hematoma or lobectomy (n, %) (*)	42	16.7	28	15.7	14	18.9	0.5362	34	18.9	8	11.1	0.1345
Decompressive craniectomy (n, %) (*)	27	10.7	15	8.4	12	16.2	0.0686	21	11.7	6	8.3	0.4396
Blood transfusion before admission (n, %)	34	13.5	25	14.0	9	12.2	0.6903	26	14.4	8	11.1	0.4841
Pneumonia (n, %) (*)	29	11.5	16	9.0	13	17.6	0.0519	19	10.6	10	13.9	0.4538
Osmotherapy (n, %) (*)	112	44.4	75	42.1	37	50.0	0.2525	89	49.4	23	31.9	0.0115
GCS score ≥ 8 (n, %)	62	24.6	39	21.9	23	31.1	0.1237	37	20.6	25	34.7	0.0183
Patient age, years (mean, sd)	47.4	17.4	48.7	17.9	44.1	15.7	0.0565	50.8	16.4	38.7	16.9	0.0000
Haemoglobin, g/dL (mean, sd)	11.8	2.3	11.7	2.2	12.1	2.5	0.1824	11.8	2.4	11.9	1.9	0.7373
Platelets, counts/mm^3^ (mean, sd)	206.7	78.0	207.4	79.7	205.1	74.2	0.8312	209.0	83.8	200.9	61.1	0.4589
Serum creatinine, mmol/L (mean, sd)	71.1	29.3	71.1	27.6	71.1	33.3	0.9853	72.4	32.6	67.9	18.7	0.2732
Arterial pH (mean, sd)	7.3	0.1	7.3	0.1	7.3	0.1	0.0978	7.3	0.1	7.3	0.1	0.6317
Serum proteins, g/L (mean, sd)	58.2	10.4	57.7	10.6	59.6	9.7	0.1662	58.0	10.7	58.8	9.7	0.5963
Serum urea, mmol/L (mean, sd)	5.0	2.5	5.2	2.7	4.7	1.8	0.1827	5.2	2.3	4.5	2.9	0.0505
PaO_2_/FiO_2_ ratio (mean, sd)	302.7	174.0	292.7	154.7	326.6	212.9	0.1595	282.1	172.4	354.2	168.4	0.0028
SAPS II score (mean, sd)	47.6	11.4	47.6	10.7	47.6	12.9	0.9847	49.9	10.8	41.8	10.7	0.0000

GOS score was dichotomised into favourable outcomes (good recovery or moderate disability) or unfavourable outcomes (severe disability, vegetative state or death).

GOS, Glasgow outcome Scale; SAP, systolic arterial pressure; HICP, high intracranial pressure; GCS, Glasgow Coma Scale; PaO_2_, partial arterial pressure of oxygen; FiO_2_, fraction of inspired oxygen; SAPS, Simplified Acute Physiology Score.

*Before HICP.

For a MacBook pro with a 2.6 GHz Intel Core i7 processor (6 cores), the results were available in 6.5 min for the ELR, 16.3 min for the LLR, 7.1 min for the NN, 2.3 min for the BCART, 2.6 min for the SVM, and 7 min for the SL.

## Discussion

When modelling the outcome model for the GC in the presence of small to medium sample sizes, the results of our simulations tended to demonstrate that ML techniques allow for accurate estimations of causal effects. Overall, the SL remained robust in all situations and achieved a relevant compromise between both bias reduction and variance estimation. In contrast, the performances of the other methods tended to vary more significantly according to the complexity of the relationships between the covariates and the outcome (simplistic versus realistic situations) and the sample size. Nevertheless, in some situations, the other methods obtained better performances than those of the SL. When the sample size was small ($n$ = 100) in the realistic scenario, the SVM had a larger MB but a smaller ASD, with an overall smaller RMSE. In this situation, the two ML techniques (SL and SVM) were even associated with lower variances than that of the perfectly specified LR. For instance, the variance bias was − 3.7% for the SL versus 10.7% for the perfectly specified LR. One can explain this result by the sample-to-sample fluctuation, which can lead to an observed structure that is different from the theoretical one. When the sample size was small in the simplistic scenario, the penalized methods (ELR and LLR) had lower MBs and similar RMSEs.

The use of ML techniques for causal inference does not preclude human intervention. In addition to the choice of the Q-model, we need to exclude the mediators, colliders and instrumental variables by considering the underlying causal structure. The use of directed acyclic graphs can help with this task^33^. We also emphasise that ML techniques do not serve as a cure-all for poor study designs or poor data quality. It is of primary importance to investigate the identifiability conditions: the exposure levels correspond to well-defined interventions, the corresponding conditional probabilities depend only on the measured covariates, and must be higher than zero. These assumptions are consistency, exchangeability, and positivity, respectively^34^. In this paper, we focused on the estimation of a causal effect given that the identifiability conditions were satisfied. In practice, the predictive performance of the Q-model is not sufficient to ensure the absence of bias in the estimation of the causal effect, which requires a precise conceptual knowledge of the causal model^35^.

Perfect knowledge of the causal structure is impossible to obtain in practice. Therefore, the analyst and the investigator construct the Q-model to approximate the causal structure as closely as possible. This may involve different steps such as the transformation of the continuous covariates to respect the log-linearity assumption, the selection of the covariates, or the choice of relevant interaction(s). While the steps performed by the analyst are data-driven and stochastic, they are systematically ignored in the estimation of the effect variance^36^. The widespread interest in (human-free) ML stems from the possibility of considering a valid post-selection inference by bootstrapping the entire estimation procedure^29^.

ML techniques are often associated with big data, especially in the field of causal inference^8,37,38^. Nevertheless, we described the acceptable properties of the SL used in a GC framework to provide causal inference conclusions from databases including several hundred subjects. To obtain this result, we first selected several simple ML techniques. We excluded deep learning techniques, such as neural networks with multiple hidden layers. Second, we retained the ML techniques that allow for maintaining the exposure as one of the predictors. Third, we included two parametric models. Fourth, we used bootstrap cross-validation to prevent overfitting. Fifth, we used two ML techniques (NN and SVM) for which there was no selection of predictors. Consequently, all covariates were also included in the SL, even those with low contributions due to having no association. The removal of confounders in GC can result in confounding bias, which can explain the poor performances of the penalized methods in realistic situations. These choices participated in the lower bias of the SL versus that of BCART. Our GC results are in agreement with the conclusions of Gruber et al., which concerned PS-based analyses^8^. Indeed, BCART is an ensemble learning method that avoids cross-validation by a single partitioning of the data into training and validation sets. It allows us to reduce the computational time, but it should be used with caution for small sample sizes.

Our study suffered from limitations. First, the results from the simulations cannot be generalised to all situations. Even if they are consistent with the current literature related to the use of ML in PS-based analyses, theoretical arguments are missing for generalisation purposes. Second, one perspective of our work is to improve the proposed SL with additional ML techniques or differently tuned techniques. For instance, we fixed the length of the tuning grid at 20; a lower value may be acceptable for reducing the computational time. The $V$-fold cross-validation is also an important parameter. We fixed $V$ = 10, as conventionally used. A more appropriate choice could also be studied. For example, Naimi and Balzer recommended increasing $V$ as the sample size decreases^22^. Third, we focused on the comparison of the ML techniques used in GC. We did not perform comparisons with other methods used for causal inference, such as the influence function-based or doubly robust estimators. In particular, the double/debiased machine learning and targeted maximum likelihood estimator allow for the unrestricted use of data-adaptive methods^38^. The principle is to combine the modelling of the outcome and exposure mechanisms to obtain an unbiased estimate when at least one of the two models is well-specified. However, such doubly robust estimators also have several drawbacks. If both models are misspecified, the estimation is more biased than that of a single-robust estimator such as GC^14^. The inclusion of a mediator also leads to more bias than that of GC^39^. Several studies have additionally reported that GC has a lower variance than those of doubly robust estimators^11–14^. As previously stated, the use of GC also represents a partial solution for preventing the selection of instrumental variables since it is independent of the exposure modelling. Fourth, our study focused on the situation where both the exposure status and the outcome are binary. The generalisation of our approach to other contexts, especially for time-to-event outcomes, represents a short-term goal. Finally, we focused on the $ACE$ if the entire sample had been exposed and if it had not been exposed. Additional analyses are needed to confirm these results to estimate the average causal effect only for the exposed individuals^40^.

In conclusion, the super-learned G-computation is a promising method for causal inference, even with only several hundred subjects. The SVM represents an interesting alternative for small sample sizes with one hundred subjects when the relationships between the covariates and the outcome are complex. For such a small sample size, penalized methods appeared to be the best alternatives when the relationships were simplistic (few covariates with linear relationships and without interactions). The computation times of these ML techniques associated with GC were reasonable. Note that GC with the SL as the Q-model is implemented in the *RISCA* package (cran.r-project.org, version ≥ 0.82). The user can set the number of splits for cross-validation and the number of parameter combinations to be evaluated. This is a particular solution, but it is not recommended for analysing any type of data using the same algorithm. We believe that such ML techniques constitute an opportunity for analysts to save some of their time used for repetitive modelling steps and use it for applying prior knowledge of the medical field and improving their comprehension of the given data structure.

## Supplementary Information

Supplementary Information.
