# Supplementary material for: G-computation and machine learning for estimating the causal effects of binary exposure statuses on binary outcomes
Source: Sci Rep. 2021 Jan 14;11:1435. doi: 10.1038/s41598-021-81110-0 (PMC7809122; doi:10.1038/s41598-021-81110-0)

Supplementary materials of the manuscript by Le Borgne et al. entitled "G-computation and machine learning for estimating the causal effects of binary exposure statuses on binary outcomes"

Florent Le Borgne[1,2], Arthur Chatton[1,2], Maxime Léger[1,3], Rémi Lenain[1,4], and Yohann Foucher[1,5]

[1] INSERM UMR 1246 - SPHERE, Nantes University, Tours University, Nantes, France.

[2] IDBC-A2COM, Pacé, France.

[3] Département d'Anesthésie Réanimation, Centre Hospitalier Universitaire d'Angers, Angers, France.

[4] Lille University Hospital, Lille, France

[5] Nantes University Hospital, Nantes, France.

Table of contents:

Definition of the criteria reported in the simulations. 3

Table S1. Models used for simulations in the realistic situation (Figure 1A in the main text). 4

Table S2. Models used for simulations in the simplistic situation (Figure 1B in the main text). 5

Table S3. Performances of G-computation in a realistic situation with the following Q-models: the theoretical logistic regression, elasticnet logistic regression, lasso logistic regression, neural network, support vector machine, boosted CART and super learner. 6

Table S4. Performances of G-computation in a simplistic situation with the following Q-models: the theoretical logistic regression, elasticnet logistic regression, lasso logistic regression, neural network, support vector machine, boosted CART and super learner. 7

Table S5. List of the two sets of variables retained for the analysis of the case study. The set A contains all the available covariates that occurred prior to the exposure (i.e., prior to the first episode of intracranial hypertension). The set B is reduced to the variables that cause the outcome. These two sets were defined by M.L. based on his prior knowledge. 8

Figure S1. Calibration plots related to the predictions based on the lasso logistic regression in the realistic situation for 10 simulated datasets. 9

Figure S2. Calibration plots related to the predictions based on the elasticnet logistic regression in the realistic situation for 10 simulated datasets. 10

Figure S3. Calibration plots related to the predictions based on the neural network in the realistic situation for 10 simulated datasets. 11

Figure S4. Calibration plots related to the predictions based on the support vector machine in the realistic situation for 10 simulated datasets. 12

Figure S5. Calibration plots related to the predictions based on the super learner in the realistic situation for 10 simulated datasets. 13

Figure S6. Calibration plots related to the predictions based on the boosted classification and regression trees in the realistic situation for 10 simulated datasets. 14

Figure S7. Calibration plots related to the predictions based on the lasso logistic regression in the simplistic situation for 10 simulated datasets. 15

Figure S8. Calibration plots related to the predictions based on the elasticnet logistic regression in the simplistic situation for 10 simulated datasets. 16

Figure S9. Calibration plots related to the predictions based on the neural network in the simplistic situation for 10 simulated datasets. 17

Figure S10. Calibration plots related to the predictions based on the support vector machine in the simplistic situation for 10 simulated datasets. 18

Figure S11. Calibration plots related to the predictions based on the super learner in the simplistic situation for 10 simulated datasets. 19

Figure S12. Calibration plots related to the predictions based on the boosted classification and regression trees in the simplistic situation for 10 simulated datasets. 20

Definition of the criteria reported in the simulations.

Let $\hat{\theta}_k$ and $\widehat{sd}(\hat{\theta}_k)$ the average causal effect (ACE) and its standard deviation estimated in the kth simulated data set and $\bar{\theta}$ the true ACE $\bar{\theta}$ ($k = 1, \dots, 1000$). The criteria used in our simulation study are the following:

i) The mean bias (MB) : $\frac{1}{1000}\sum_{k=1}^{1000}(\hat{\theta}_k - \bar{\theta}) * 100$

ii) The root mean square error (RMSE) : $\sqrt{\frac{1}{1000}\sum_{k=1}^{1000}(\hat{\theta}_k - \bar{\theta})^2}$

iii) The empirical standard deviation (ESD): $\sqrt{\frac{1}{999}\sum_{k=1}^{1000}(\hat{\theta}_k - \bar{\theta})^2}$

iv) The asymptotic standard deviation (ASD): $\frac{1}{1000}\sum_{k=1}^{1000}\widehat{sd}(\hat{\theta}_k)$

v) The variance estimation bias: $100 * (ASD - ESD)/ESD$

vi) The empirical coverage rate of the nominal 95% confidence interval (95%CI): $\frac{1}{1000}\sum_{k=1}^{1000}\left(\mathrm{I}\left(95\%CI_{inf,k} \leq \theta \leq 95\%CI_{sup,k}\right)\right)$, where $95\%CI_{inf,k}$ and $95\%CI_{sup,k}$ are the lower and upper bounds of the 95%CI estimated by bootstrap in the kth simulated data set, respectively.

vii) The statistical power: $\frac{1}{1000}\sum_{k=1}^{1000}\left(\mathrm{I}\left(95\%CI_{inf,k} > 0\right) + \mathrm{I}\left(95\%CI_{sup,k} < 0\right)\right)$

Table S1. Models used for simulations in the realistic situation (Figure 1A in the main text).

Distribution	Linear predictor
X_1 ~ Gaussian	0
X_2 ~ Gaussian	$\beta_0 + \beta_1 * X_1$
X_3 ~ Gaussian	$\beta_0 - \beta_1 * X_1 - \beta_1 * X_2$
X_4 ~ Gaussian	$\beta_0 + \beta_1 * X_3$
X_5 ~ Gaussian	0
$\tilde{X}_6$ ~ Gaussian	0
X_6 ~ Bernouilli	1 if $\tilde{X}_6$ > 0.66 and 0 otherwise (prevalence ~ 25%)
$\tilde{X}_7$ ~ Gaussian	$\beta_0 - \beta_1 * X_5$
X_7 ~ Bernouilli	1 if $\tilde{X}_7$ > -0.40 and 0 otherwise (prevalence ~ 40%)
X_8 ~ Gaussian	$\beta_0 - \beta_1 * X_6$
$\tilde{X}_9$ ~ Gaussian	$\beta_0 + \beta_1 * X_7$
X_9 ~ Bernouilli	1 if $\tilde{X}_9$ > -0.80 and 0 otherwise (prevalence ~ 75%)
X_{10} ~ Gaussian	$\beta_0 + \beta_1 * X_8$
X_{11} ~ Gaussian	0
$\tilde{X}_{12}$ ~ Gaussian	$\beta_0 + \beta_1 * X_9$
X_{12} ~ Bernouilli	1 if $\tilde{X}_{12}$ > 0.84 and 0 otherwise (prevalence ~ 25%)
$\tilde{X}_{13}$ ~ Gaussian	$\beta_0 + \beta_1 * X_{10}$
X_{13} ~ Bernouilli	1 if $\tilde{X}_{13}$ > -0.09 and 0 otherwise (prevalence ~ 50%)
X_{14} ~ Gaussian	$\beta_0 - \beta_1 * X_{12} - \beta_1 * X_{11}$
X_{15} ~ Gaussian	$\beta_0 - \beta_1 * X_{12}$
$\tilde{X}_{16}$ ~ Gaussian	0
X_{16} ~ Bernouilli	1 if $\tilde{X}_{16}$ > -0.66 and 0 otherwise (prevalence ~ 75%)
$\tilde{X}_{17}$ ~ Gaussian	$\beta_0 - \beta_1 * X_{16}$
X_{17} ~ Bernouilli	1 if $\tilde{X}_{17}$ > -0.92 and 0 otherwise (prevalence ~ 50%)
X_{18} ~ Gaussian	0
$\tilde{X}_{19}$ ~ Gaussian	0
X_{19} ~ Bernouilli	1 if $\tilde{X}_{19}$ > 0.66 and 0 otherwise (prevalence ~ 25%)
$\tilde{X}_{20}$ ~ Gaussian	0
X_{20} ~ Bernouilli	1 if $\tilde{X}_{20}$ > 0.66 and 0 otherwise (prevalence ~ 25%)
X_{21} ~ Gaussian	0
$\tilde{X}_{22}$ ~ Gaussian	0
X_{22} ~ Bernouilli	1 if $\tilde{X}_{22}$ > 0.66 and 0 otherwise (prevalence ~ 25%)
Z ~ Bernouilli	$\beta_0 + \beta_1 * X_1 - \beta_1 * X_3 + \beta_1 * X_5 - \beta_1 * X_7 + \beta_1 * X_9 - \beta_1 * X_{11} + \beta_1 * X_{13} - \beta_1 * X_{15} - \beta_1 * X_{17} + \beta_1 * X_{19} - \beta_1 * X_{21}$
Y ~ Bernouilli	$-1.1 + \beta_1 * I(X_2 > -0.40) - \beta_1 * X_3 + (\beta_1/2) * X_3^2 + \beta_1 * X_6 + \beta_1 * X_7 + \beta_1 * X_{10} + \beta_1 * 0.5 * X_{11}^2 - \beta_1 * X_{14} - \beta_1 * I(X_{15} > -0.57) + \beta_1 * X_{18} + \beta_1 * X_{19} + \beta_1 * Z + \beta_1 * 0.5 * Z * X_{18}$

For the Gaussian distributions, the standard errors were 1 and the link function with the linear predictor was the identity function. For the Bernoulli distribution, the link function with the linear predictor was the logit function. $\beta_0 = -0.4$, $\beta_1 = log(2.00)$, $I(a) = 1$ if a is true and 0 otherwise.

Table S2. Models used for simulations in the simplistic situation (Figure 1B in the main text).

Distribution	Linear predictor
X_1 ~ Bernouilli	0
X_2 ~ Bernouilli	0
X_3 ~ Gaussian	0
X_4 ~ Bernouilli	0
X_5 ~ Bernouilli	0
X_6 ~ Gaussian	0
X_7 ~ Bernouilli	0
X_8 ~ Bernouilli	0
X_9 ~ Gaussian	0
Z ~ Bernouilli	$-0.8 + \beta_2 * X_1 + \beta_1 * X_2 - \beta_2 * X_4 - \beta_1 * X_5 + \beta_2 * X_7 + \beta_1 * X_8$
Y ~ Bernouilli	$-0.8 + \beta_Z * Z + \beta_2 * X_1 - \beta_2 * X_2 - \beta_2 * X_3 + \beta_1 * X_4 - \beta_1 * X_5 + \beta_1 * X_6$

For the Gaussian distributions, the standard errors were 1 and the link function with the linear predictor was the identity function. For the Bernoulli distribution, the link function with the linear predictor was the logit function. $\beta_1 = log(1.50)$, $\beta_2 = log(3.00)$, and $\beta_Z = log(1.75)$.

Table S3. Performances of G-computation in a realistic situation with the following Q-models: the theoretical logistic regression, elasticnet logistic regression, lasso logistic regression, neural network, support vector machine, boosted CART and super learner.

n (EPV)	Method	RMSE	MB (%)	ESD	ASD	VEB (%)	Cover (%)	Power (%)
100 (2.7)	Perfectly specified logistic regression	0.092	0.422	0.092	0.102	10.7	95.8	12.9
	Elasticnet logistic regression	0.117	5.751	0.102	0.096	-5.1	88.7	33.5
	Lasso logistic regression	0.113	5.051	0.101	0.101	0.4	91.3	28.6
	Neural network	0.069	-4.286	0.054	0.059	8.8	86.6	12.4
	Support vector machine	0.059	-0.648	0.059	0.055	-6.4	92.6	36.5
	Boosted CART	0.065	-5.114	0.039	0.017	-56.3	63.8	11.3
	Super learner	0.071	0.425	0.071	0.068	-3.7	93.1	30.8
500 (13.6)	Perfectly specified logistic regression	0.039	-0.036	0.039	0.038	-2.1	94.1	65.5
	Elasticnet logistic regression	0.047	1.700	0.044	0.043	-2.5	92.1	70.4
	Lasso logistic regression	0.045	1.398	0.043	0.043	-0.1	93.1	68.7
	Neural network	0.046	-2.075	0.041	0.048	19.0	96.1	29.5
	Support vector machine	0.043	1.365	0.041	0.041	-0.5	93.7	74.6
	Boosted CART	0.056	-4.896	0.028	0.026	-6.3	57.2	31.9
	Super learner	0.040	0.142	0.040	0.040	-1.6	95.2	65.0
1000 (27.3)	Perfectly specified logistic regression	0.027	-0.017	0.027	0.027	-0.9	94.9	93.6
	Elasticnet logistic regression	0.032	0.936	0.030	0.030	-0.7	92.2	92.4
	Lasso logistic regression	0.032	0.801	0.031	0.030	-1.3	92.9	91.5
	Neural network	0.036	-1.774	0.031	0.041	31.1	98.3	41.9
	Support vector machine	0.035	1.456	0.032	0.032	0.4	91.4	91.7
	Boosted CART	0.047	-4.017	0.241	0.023	-2.4	61.8	54.8
	Super learner	0.031	0.485	0.031	0.031	1.2	94.6	89.3

Abbreviations: MB = mean bias; RMSE = root mean square error; ESD = empirical standard deviation; ASD = asymptotic standard deviation; VEB = variance estimation bias; EPV = events per variable.

Table S4. Performances of G-computation in a simplistic situation with the following Q-models: the theoretical logistic regression, elasticnet logistic regression, lasso logistic regression, neural network, support vector machine, boosted CART and super learner.

n (EPV)	Method	RMSE	MB (%)	ESD	ASD	VEB (%)	Cover (%)	Power (%)
100 (4.1)	Perfectly specified logistic regression	0.093	-0.382	0.093	0.094	0.8	94.6	18.5
	Elasticnet logistic regression	0.096	0.293	0.096	0.097	1.0	93.9	20.2
	Lasso logistic regression	0.096	0.081	0.096	0.100	4.5	94.6	18.0
	Neural network	0.087	-1.607	0.086	0.103	19.7	96.6	10.9
	Support vector machine	0.078	-2.309	0.075	0.075	0.7	93.8	20.1
	Boosted CART	0.075	-3.874	0.065	0.061	-6.3	82.2	18.6
	Super learner	0.084	-1.255	0.083	0.083	-0.6	94.1	20.0
500 (20.4)	Perfectly specified logistic regression	0.041	-0.258	0.041	0.040	-1.8	93.6	66.6
	Elasticnet logistic regression	0.041	0.079	0.041	0.041	-1.2	94.5	67.5
	Lasso logistic regression	0.041	-0.052	0.041	0.041	0.1	94.8	65.1
	Neural network	0.043	-1.143	0.042	0.057	36.4	98.5	28.3
	Support vector machine	0.041	-0.253	0.041	0.042	3.4	94.3	64.1
	Boosted CART	0.055	-4.327	0.034	0.030	-11.0	69.6	36.5
	Super learner	0.040	-0.338	0.040	0.042	4.9	95.6	63.9
1000 (40.8)	Perfectly specified logistic regression	0.028	0.132	0.028	0.028	1.3	94.7	94.3
	Elasticnet logistic regression	0.028	0.243	0.028	0.029	1.8	95.1	93.8
	Lasso logistic regression	0.029	0.164	0.028	0.029	2.0	95.2	93.1
	Neural network	0.033	-1.184	0.031	0.038	23.2	97.4	62.8
	Support vector machine	0.030	0.233	0.030	0.031	4.0	95.8	89.9
	Boosted CART	0.043	-3.316	0.027	0.025	-8.2	73.1	70.7
	Super learner	0.029	-0.056	0.029	0.030	5.0	96.3	90.0

Abbreviations: MB = mean bias; RMSE = root mean square error; ESD = empirical standard deviation; ASD = asymptotic standard deviation; VEB = variance estimation bias; EPV = events per variable.

Table S5. List of the two sets of variables retained for the analysis of the case study. The set A contains all the available covariates that occurred prior to the exposure (i.e., prior to the first episode of intracranial hypertension). The set B is reduced to the variables that cause the outcome. These two sets were defined by M.L. based on his prior knowledge.

	**Set A: variables included in the machine learning techniques**	**Set B: variables included in the investigator-based logistic regression**
Female patient	X	X
Diabetes	X	X
Nosological entity: Severe trauma	X	X
SAP ≤ 90 mmHg before admission	X	X
Evacuation of subdural or extradural hematoma (*)	X	
External ventricular drain	X	
Evacuation of cerebral hematoma or lobectomy (*)	X	X
Decompressive craniectomy (*)	X	
Blood transfusion before admission	X	
Pneumonia before increased HICP	X	
Osmotherapy (*)	X	X
GCS score ≥ 8	X	X
Patient age	X	X
Hemoglobin	X	
Platelets	X	
Serum creatinine	X	
Arterial pH	X	
Serum proteins	X	
Serum urea	X	X
PaO_2/FiO_2 ratio	X	X
SAPS II score	X	

GOS score was dichotomised into favourable outcomes (good recovery or moderate disability) or unfavourable outcomes (severe disability, vegetative state or death). Abbreviations: GOS: Glasgow Outcome Scale, SAP: Systolic Arterial Pressure, HICP: High Intracranial Pressure, GCS: Glasgow Coma Scale, PaO_2: arterial partial Pressure of Oxygen, FiO_2: Fraction of Inspired Oxygen, and SAPS: Simplified Acute Physiology Score. (*) Before HICP

Figure S1. Calibration plots related to the predictions based on the lasso logistic regression in the realistic situation for 10 simulated datasets.

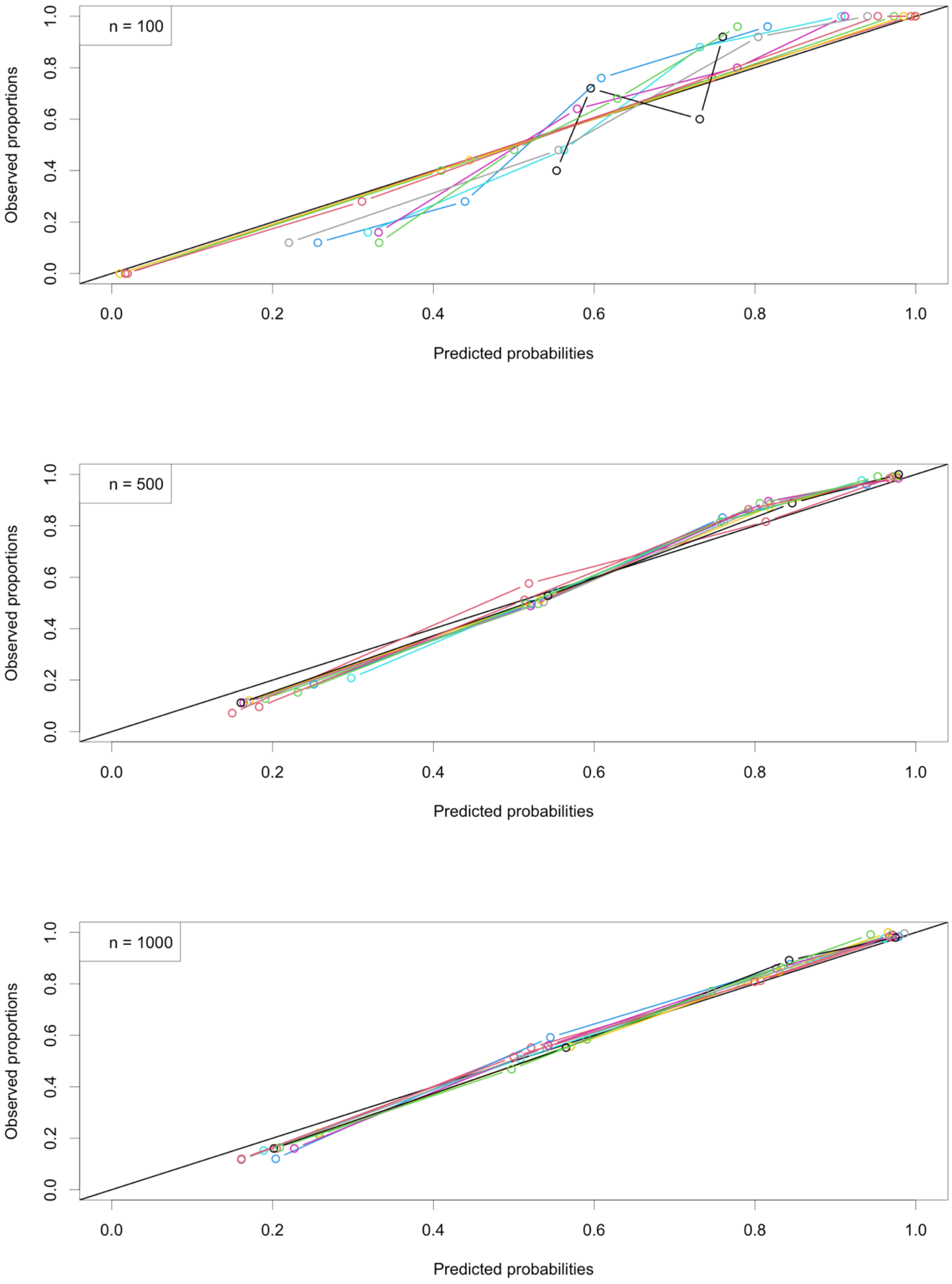

Figure S2. Calibration plots related to the predictions based on the elasticnet logistic regression in the realistic situation for 10 simulated datasets.

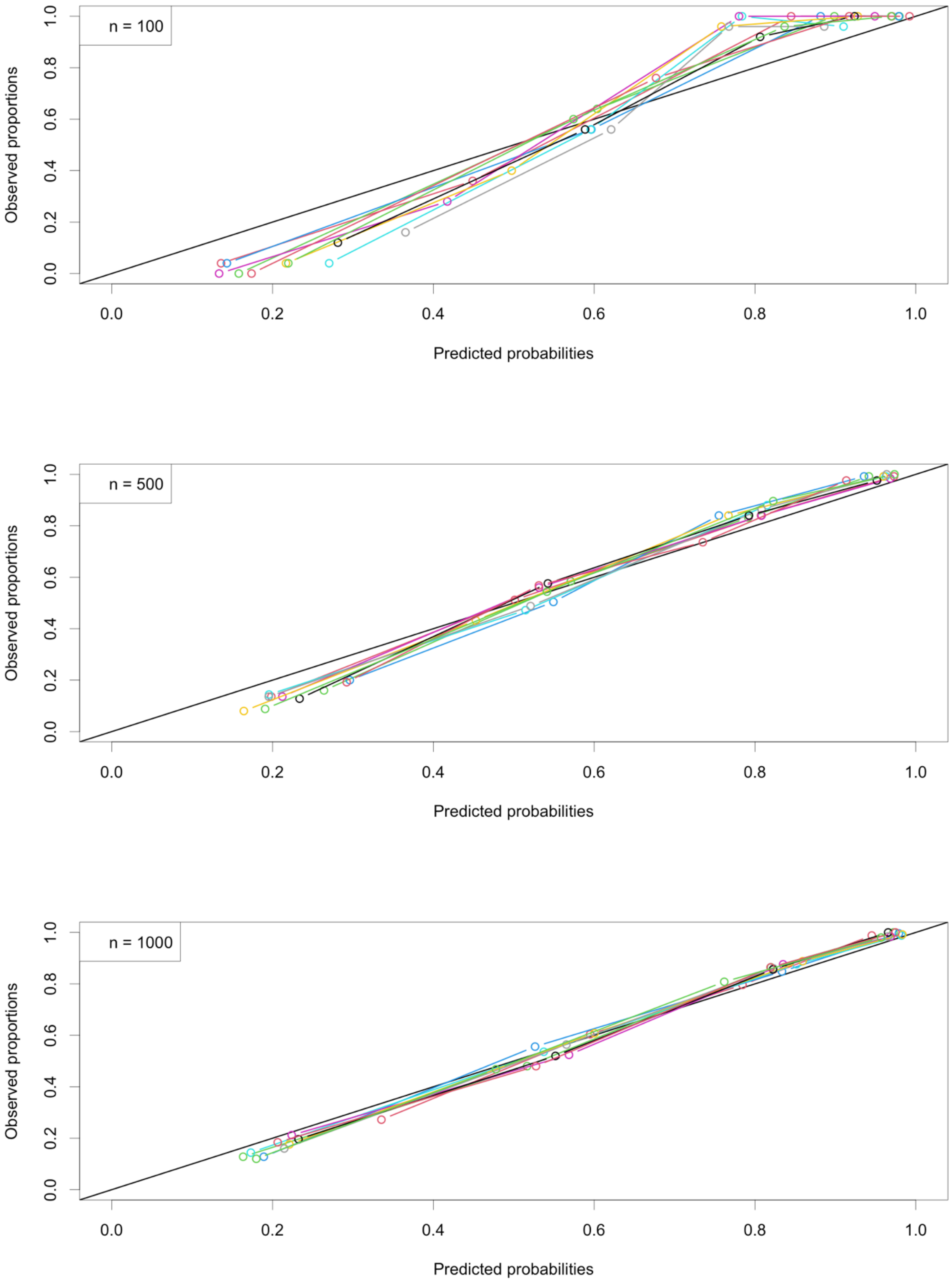

Figure S3. Calibration plots related to the predictions based on the neural network in the realistic situation for 10 simulated datasets.

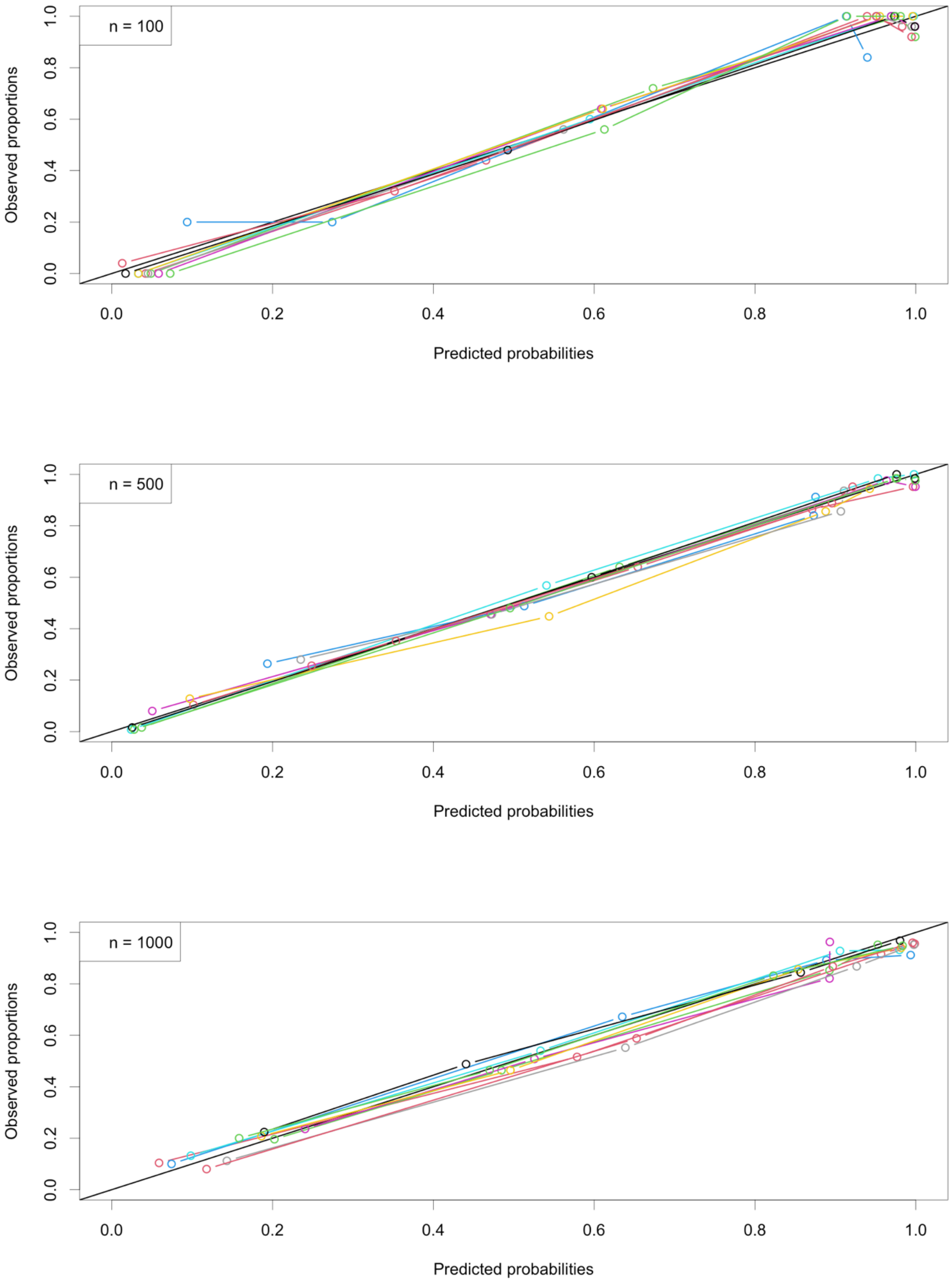

Figure S4. Calibration plots related to the predictions based on the support vector machine in the realistic situation for 10 simulated datasets.

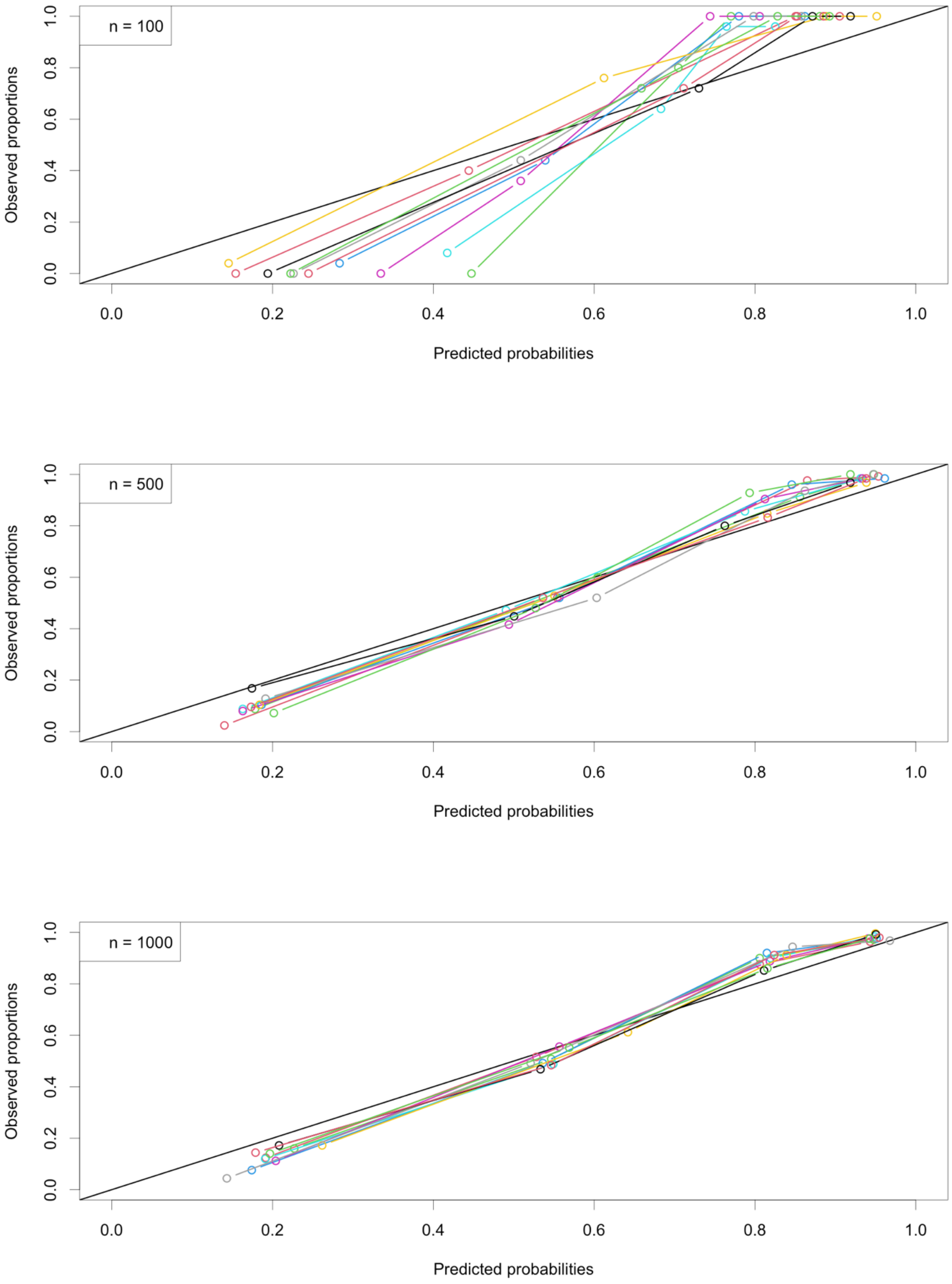

Figure S5. Calibration plots related to the predictions based on the super learner in the realistic situation for 10 simulated datasets.

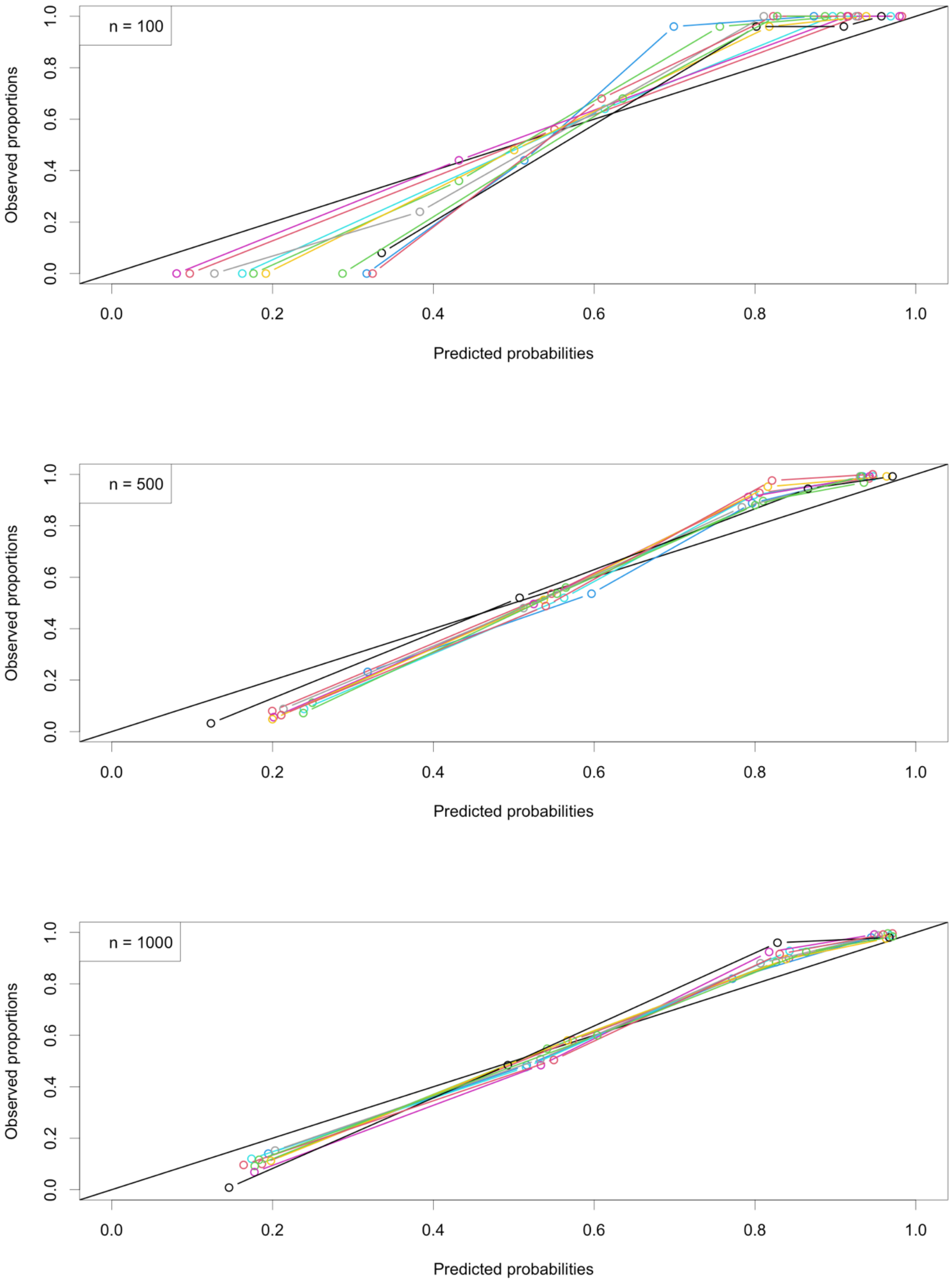

Figure S6. Calibration plots related to the predictions based on the boosted classification and regression trees in the realistic situation for 10 simulated datasets.

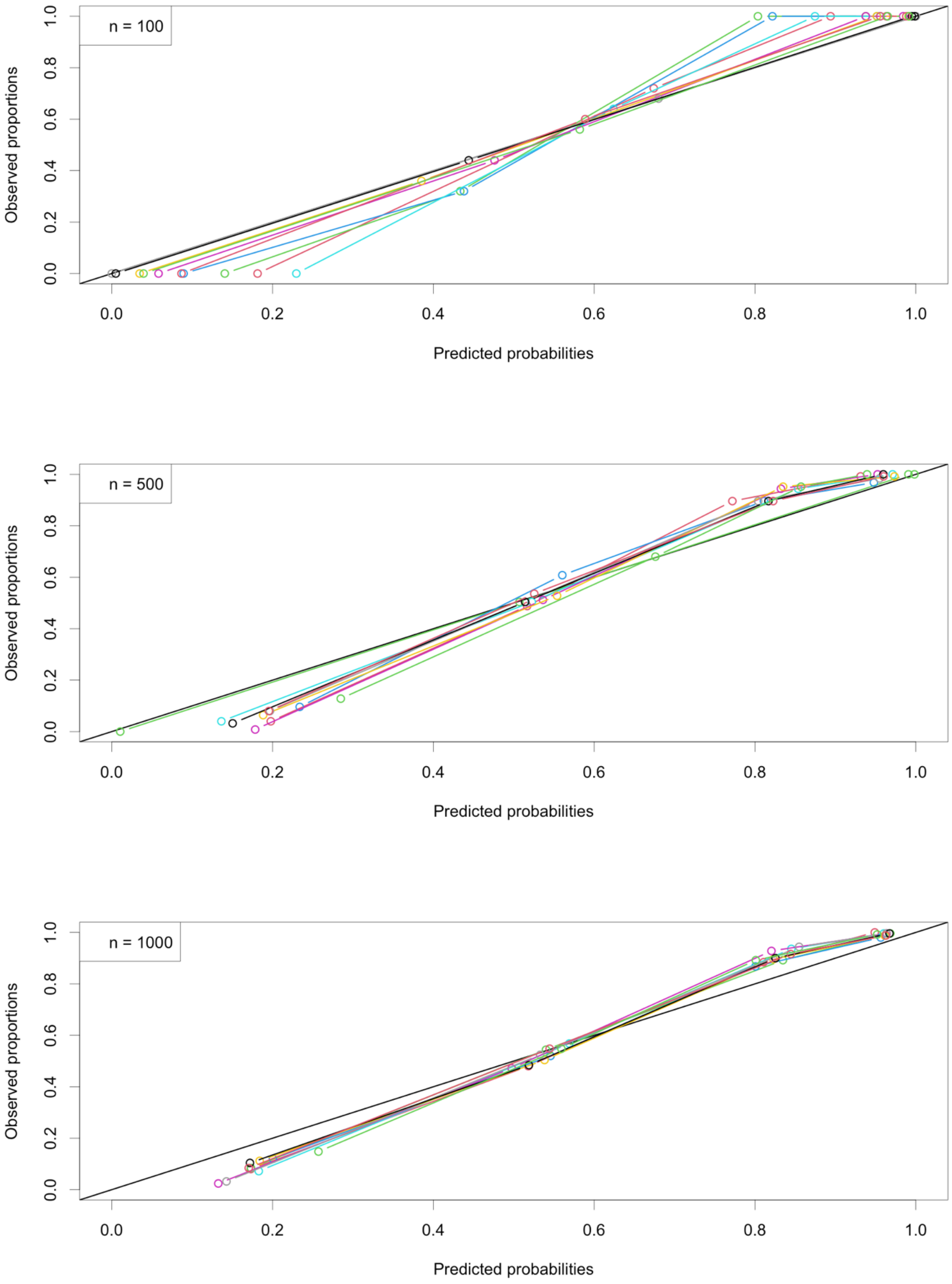

Figure S7. Calibration plots related to the predictions based on the lasso logistic regression in the simplistic situation for 10 simulated datasets.

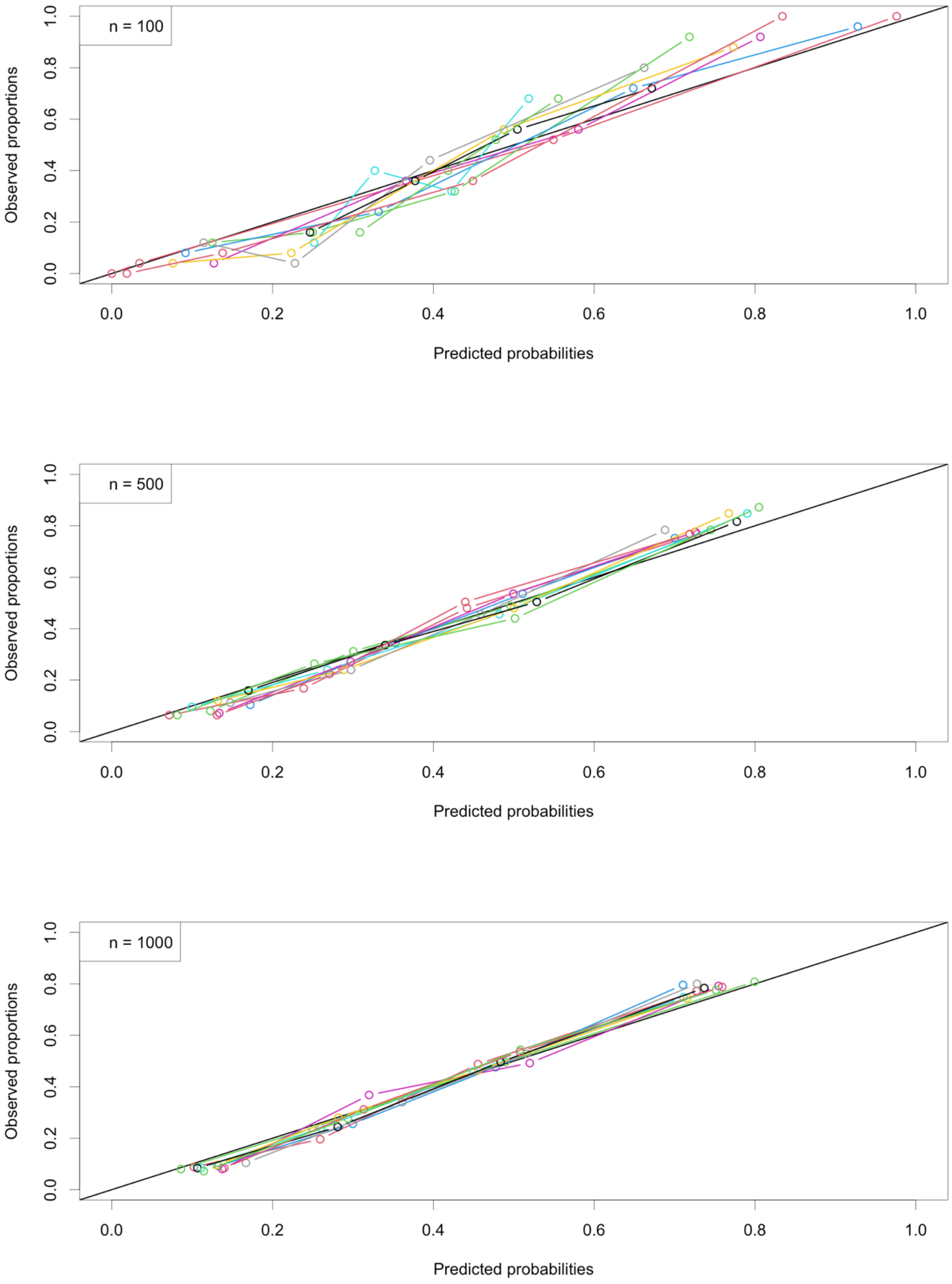

Figure S8. Calibration plots related to the predictions based on the elasticnet logistic regression in the simplistic situation for 10 simulated datasets.

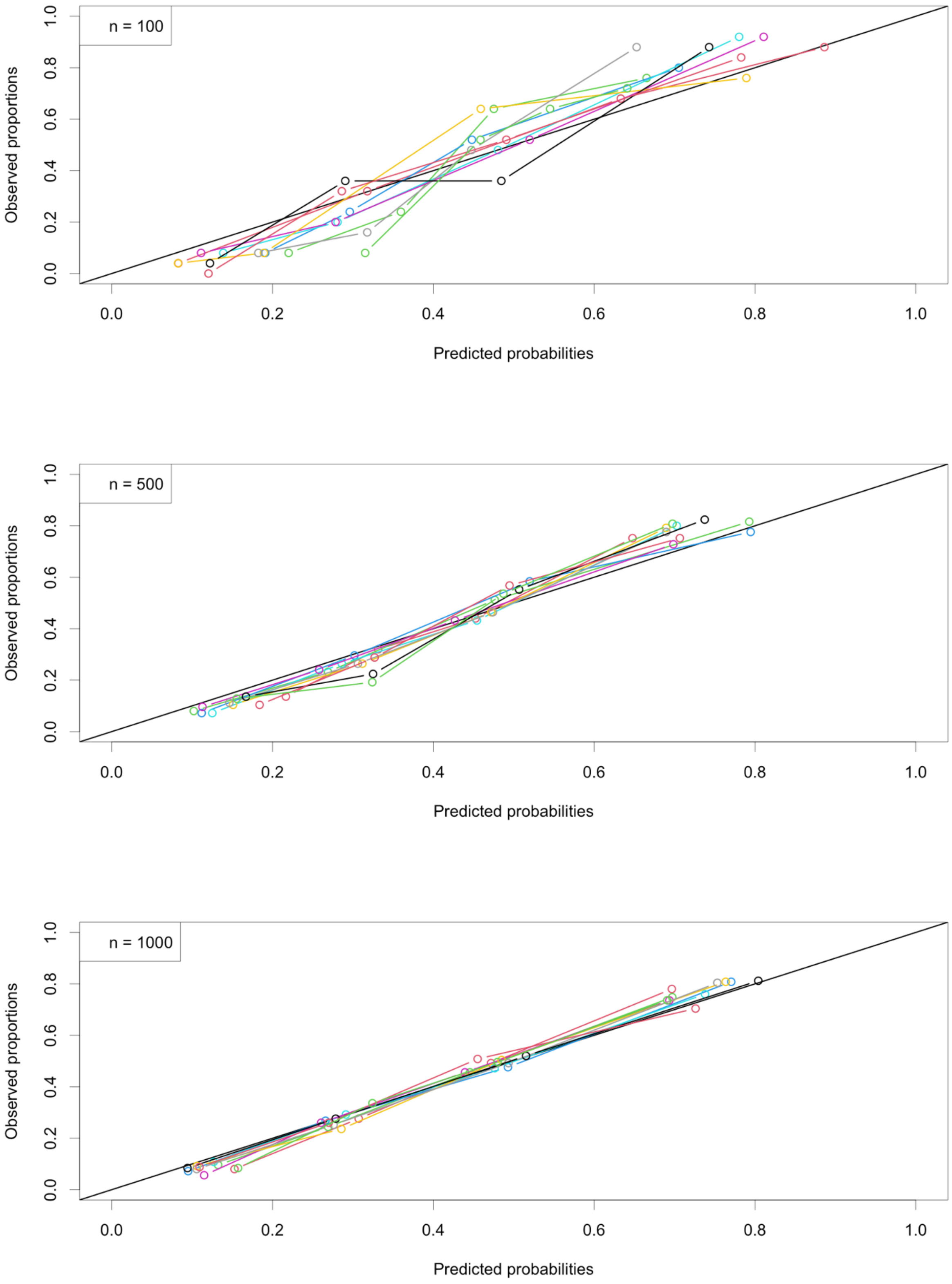

Figure S9. Calibration plots related to the predictions based on the neural network in the simplistic situation for 10 simulated datasets.

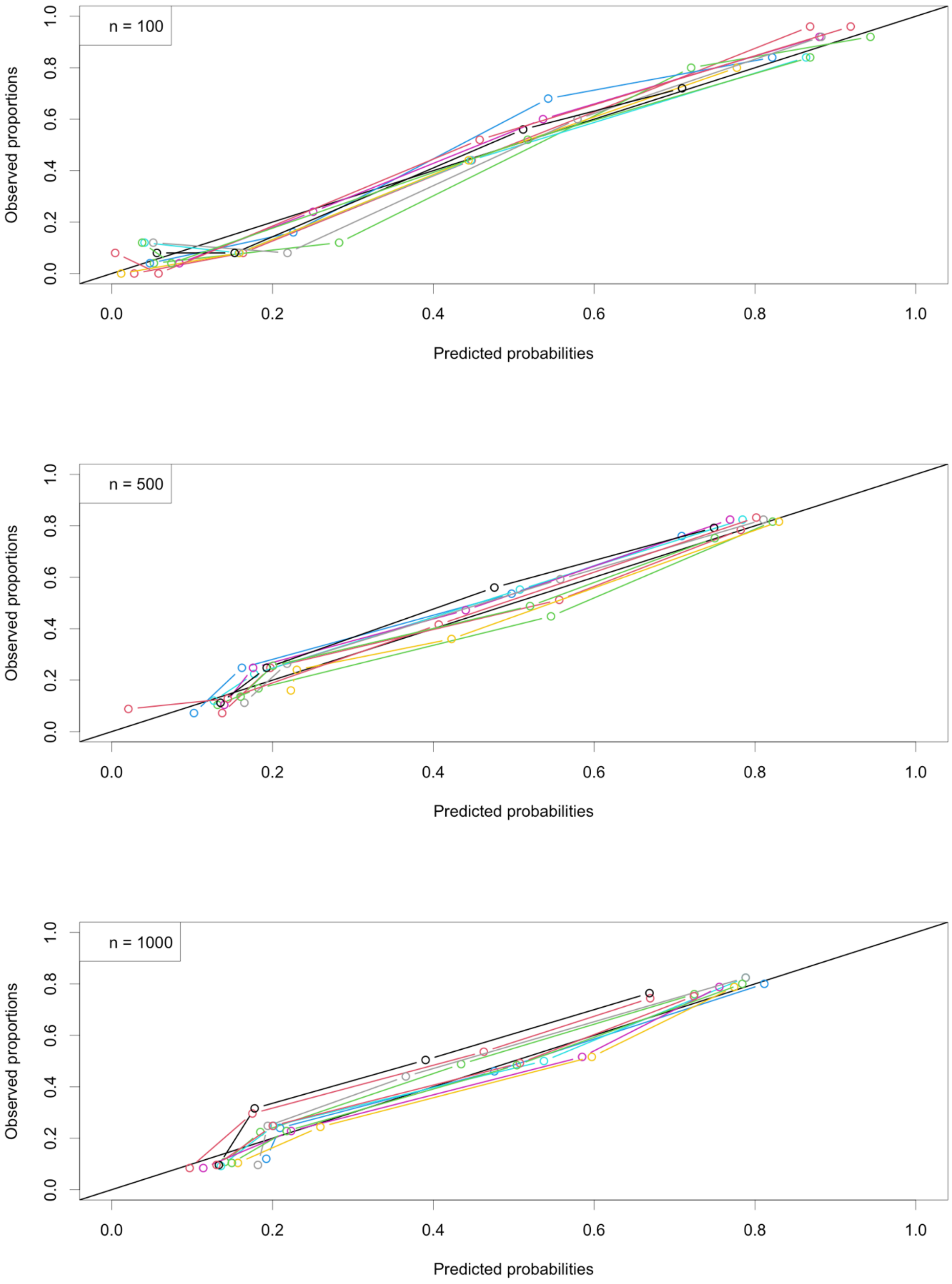

Figure S10. Calibration plots related to the predictions based on the support vector machine in the simplistic situation for 10 simulated datasets.

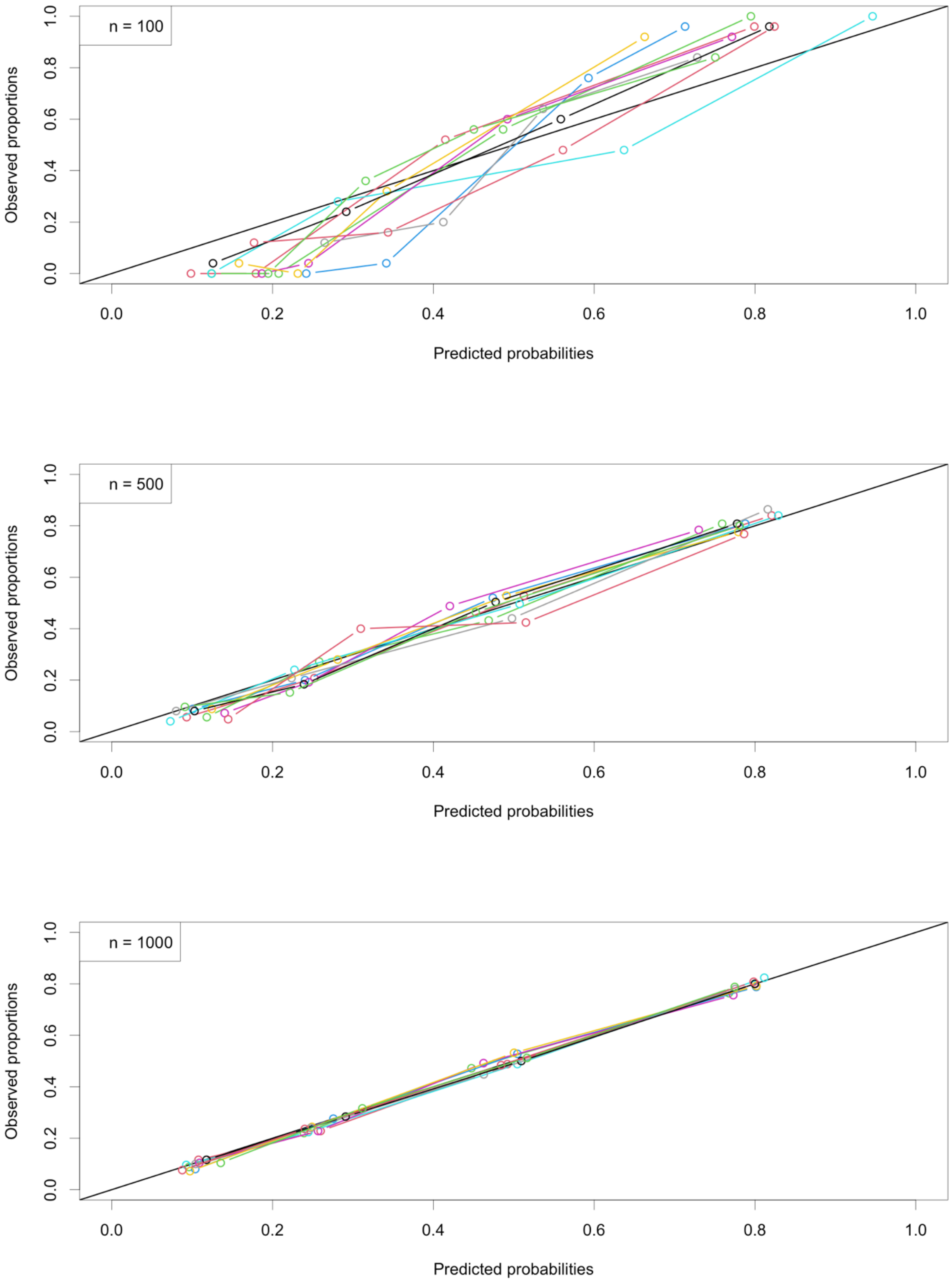

Figure S11. Calibration plots related to the predictions based on the super learner in the simplistic situation for 10 simulated datasets.

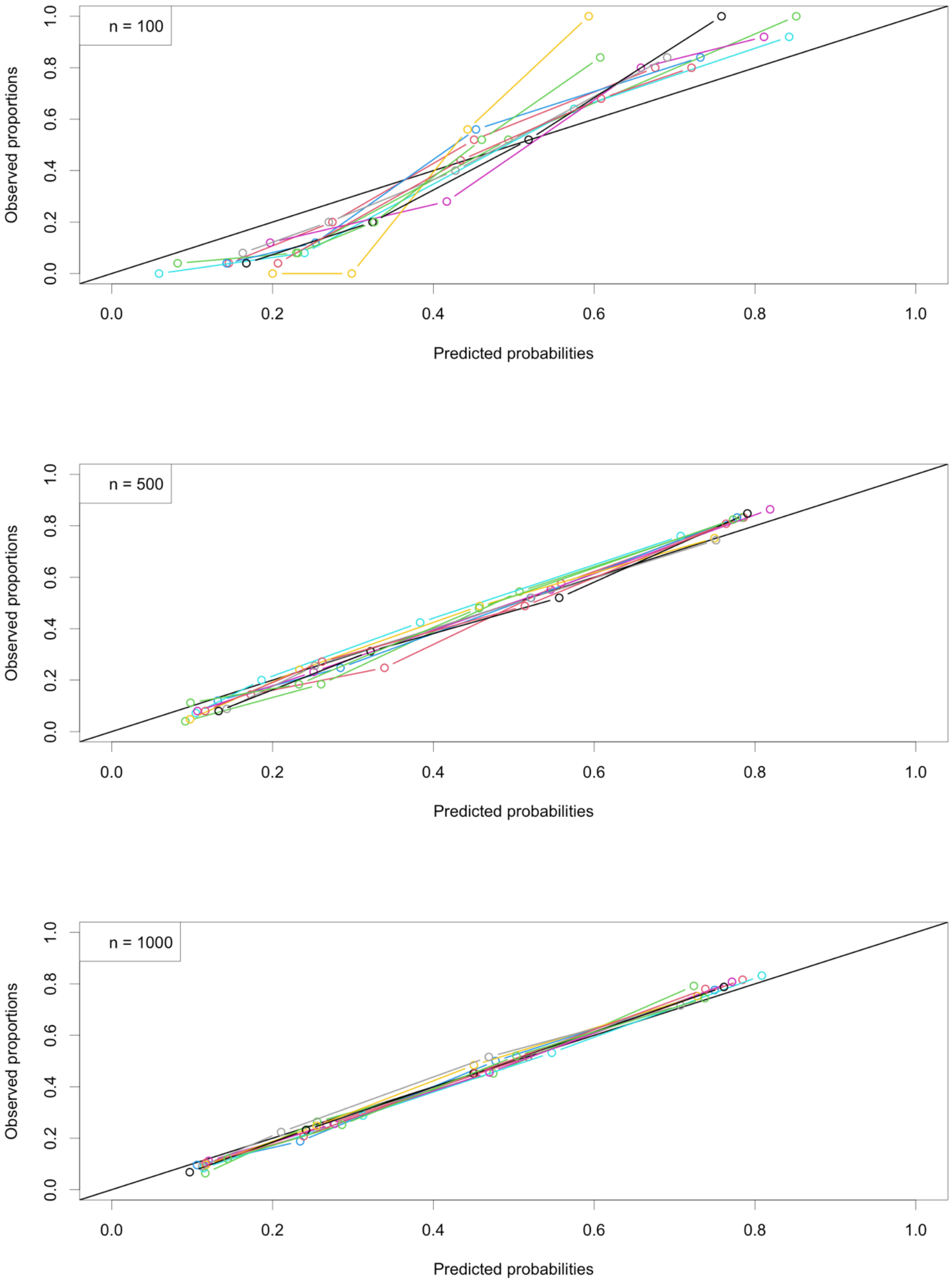

Figure S12. Calibration plots related to the predictions based on the boosted classification and regression trees in the simplistic situation for 10 simulated datasets.

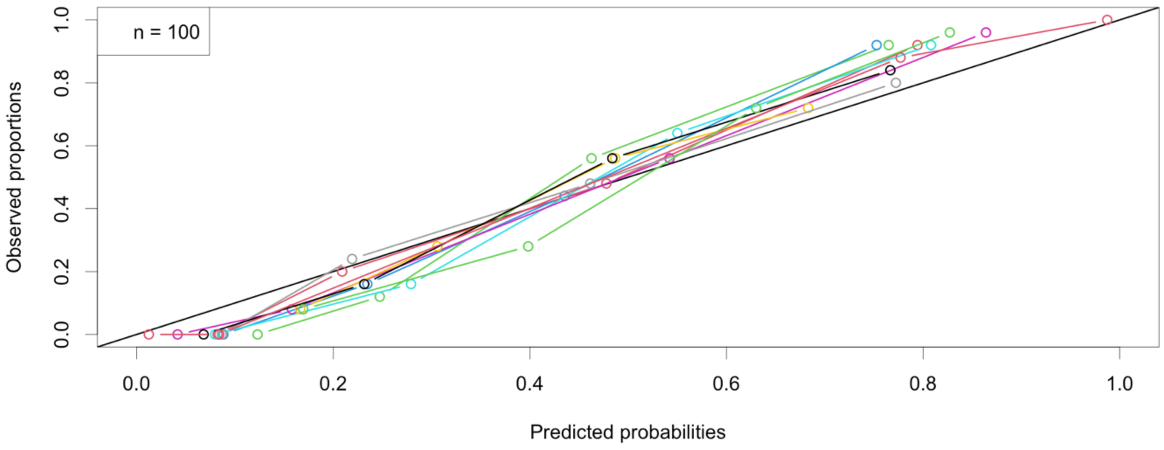

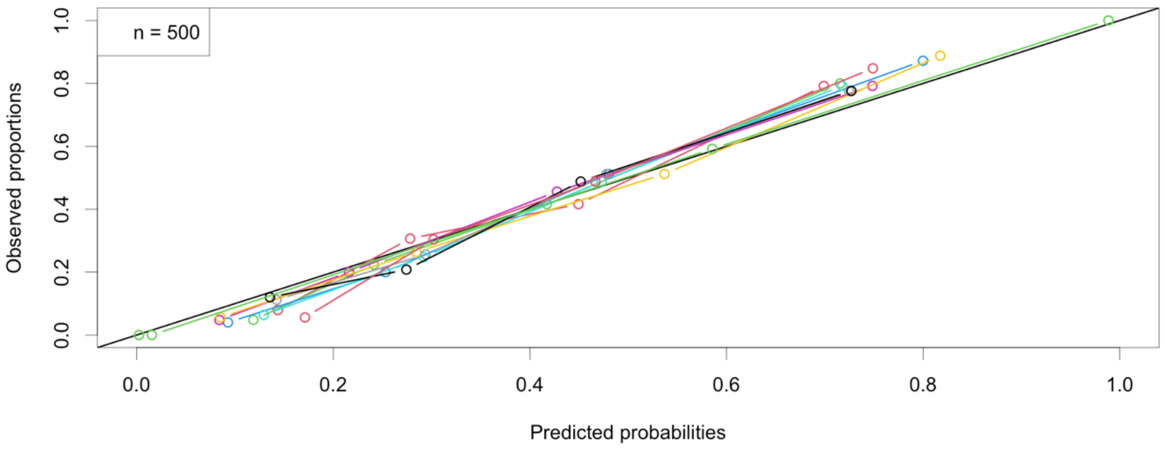

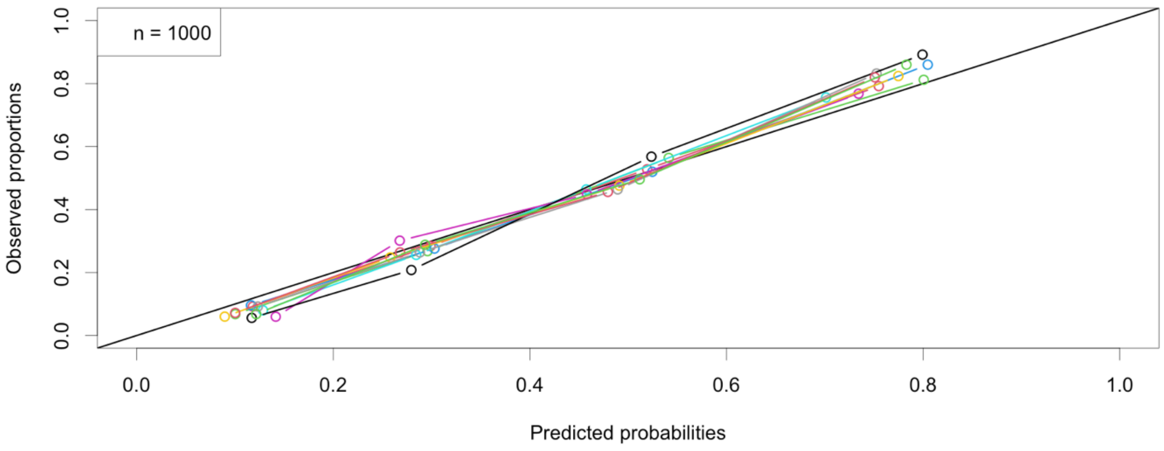

Supplement: Supplementary file 1 — Supplementary Information. [file 41598_2021_81110_MOESM1_ESM.pdf]
